# Identification and validation of a novel signature based on macrophage marker genes for predicting prognosis and drug response in kidney renal clear cell carcinoma by integrated analysis of single cell and bulk RNA sequencing

**DOI:** 10.18632/aging.205671

**Published:** 2024-03-20

**Authors:** Xiaoxu Chen, Zheyu Zhang, Zheng Qin, Xiao Zhu, Kaibin Wang, Lijuan Kang, Changying Li, Haitao Wang

**Affiliations:** 1 Department of Oncology, Tianjin Institute of Urology, The Second Hospital of Tianjin Medical University, Tianjin, China; 2 Tianjin Key Laboratory of Precision Medicine for Sex Hormones and Diseases (in Preparation), The Second Hospital of Tianjin Medical University, Tianjin, China

**Keywords:** single-cell RNA-seq, bulk RNA-seq, macrophage marker genes, signature, kidney renal clear cell carcinoma, drug response

## Abstract

Macrophages are found in a variety of tumors and play a critical role in shaping the tumor microenvironment, affecting tumor progression, metastasis, and drug resistance. However, the clinical relevance of marker genes associated with macrophage in kidney renal clear cell carcinoma (KIRC) has yet to be documented. In this study, we initiated a thorough examination of single-cell RNA sequencing (scRNA-seq) data for KIRC retrieved from the Gene Expression Omnibus (GEO) database and determined 244 macrophage marker genes (MMGs). Univariate analysis, LASSO regression, and multivariate regression analysis were performed to develop a five-gene prognostic signature in The Cancer Genome Atlas (TCGA) database, which could divide KIRC patients into low-risk (L-R) and high-risk (H-R) groups. Then, a nomogram was constructed to predict the survival rate of KIRC patients at 1, 3, and 5 years, which was well assessed by receiver operating characteristic curve (ROC), calibration curve, and decision curve analyses (DCA). Functional enrichment analysis showed that immune-related pathways (such as immunoglobulin complex, immunoglobulin receptor binding, and cytokine-cytokine receptor interaction) were mainly enriched in the H-R group. Additionally, in comparison to the L-R cohort, patients belonging to the H-R cohort exhibited increased immune cell infiltration, elevated expression of immune checkpoint genes (ICGs), and a higher tumor immune dysfunction and exclusion (TIDE) score. This means that patients in the H-R group may be less sensitive to immunotherapy than those in the L-R group. Finally, IFI30 was validated to increase the ability of KIRC cells to proliferate, invade and migrate *in vitro*. In summary, our team has for the first time developed and validated a predictive model based on macrophage marker genes to accurately predict overall survival (OS), immune characteristics, and treatment benefit in KIRC patients.

## INTRODUCTION

Renal cell carcinoma (RCC) ranks as the third most commonly occurring urologic malignancy and the eighth most prevalent cancer globally. It accounts for around 400,000 new cases and 175,000 deaths reported worldwide each year [1, 2]. Over the past 20 years, there has been an annual increase of 2% in the incidence of RCC worldwide [3]. KIRC is the leading pathological type of RCC, accounting for approximately 70% of all RCCs [4]. Currently, surgery remains the primary treatment for localized or locally advanced RCC. This includes options such as radical nephrectomy (RN) or partial nephrectomy (PN) [5]. The 5-year overall survival (OS) rate for these procedures is approximately 72%. However, it is concerning that more than 20 to 30 percent of patients are already experiencing metastatic symptoms at the time of diagnosis. This indicates an advanced stage of the disease. Unfortunately, the overall 5-year survival rate for these patients was only 12 percent, highlighting the challenges associated with advanced metastatic RCC. Targeted therapies and immunotherapies have been demonstrated to effectively prolong OS in patients with distant metastases or non-resectable RCC. Naturally, the therapeutic options for RCC are currently limited in their effectiveness [6]. In addition, there is a shortage of molecular biomarkers with the requisite reliability to accurately predict the prognosis of patients with KIRC and provide guidance for clinical treatment.

Tumor microenvironment (TME) is an important determinant of tumor behavior, progression, and aggressiveness and has a critical impact on patient survival and immunotherapy response [7, 8]. Macrophages are important regulators of inflammation and immune response in TME. It has been reported that macrophage infiltration was associated with poor prognosis in many solid and hematologic tumors [9]. In both progressive and non-progressive tumors, M1-type macrophages predominate as progenitor subtypes and exhibit antitumor activity. Conversely, in malignant and advanced tumors, tumor associated macrophages (TAMs) tend to adopt the M2 phenotype, thereby promoting tumor malignancy [10]. TAMs can produce a variety of pro-angiogenic cytokines, such as VEGF, TNF-α, and IL-8. It has been found that TAM can promote tumor progression by enhancing tumor angiogenesis in several human tumors, such as breast cancer, melanoma, glioma, bladder cancer, and prostate cancer [11]. The potential efficacy of macrophages in the treatment of KIRC needs to be further intensively investigated.

ScRNA-seq is a powerful method that allows the analysis of complex biological systems at the level of individual cells. It enables the identification of rare cell populations that are associated with tumors and metastases, thus providing insights into the heterogeneity of these processes [12]. In addition to the gene signatures comprising the five MMGs, our team also investigated the role of IFI30 in KIRC. We found that IFI30 had the highest hazard ratio (HR) in their model, indicating its potential significance in predicting patient outcomes.

## RESULTS

### Identification of macrophage marker genes

**Figure 1:**
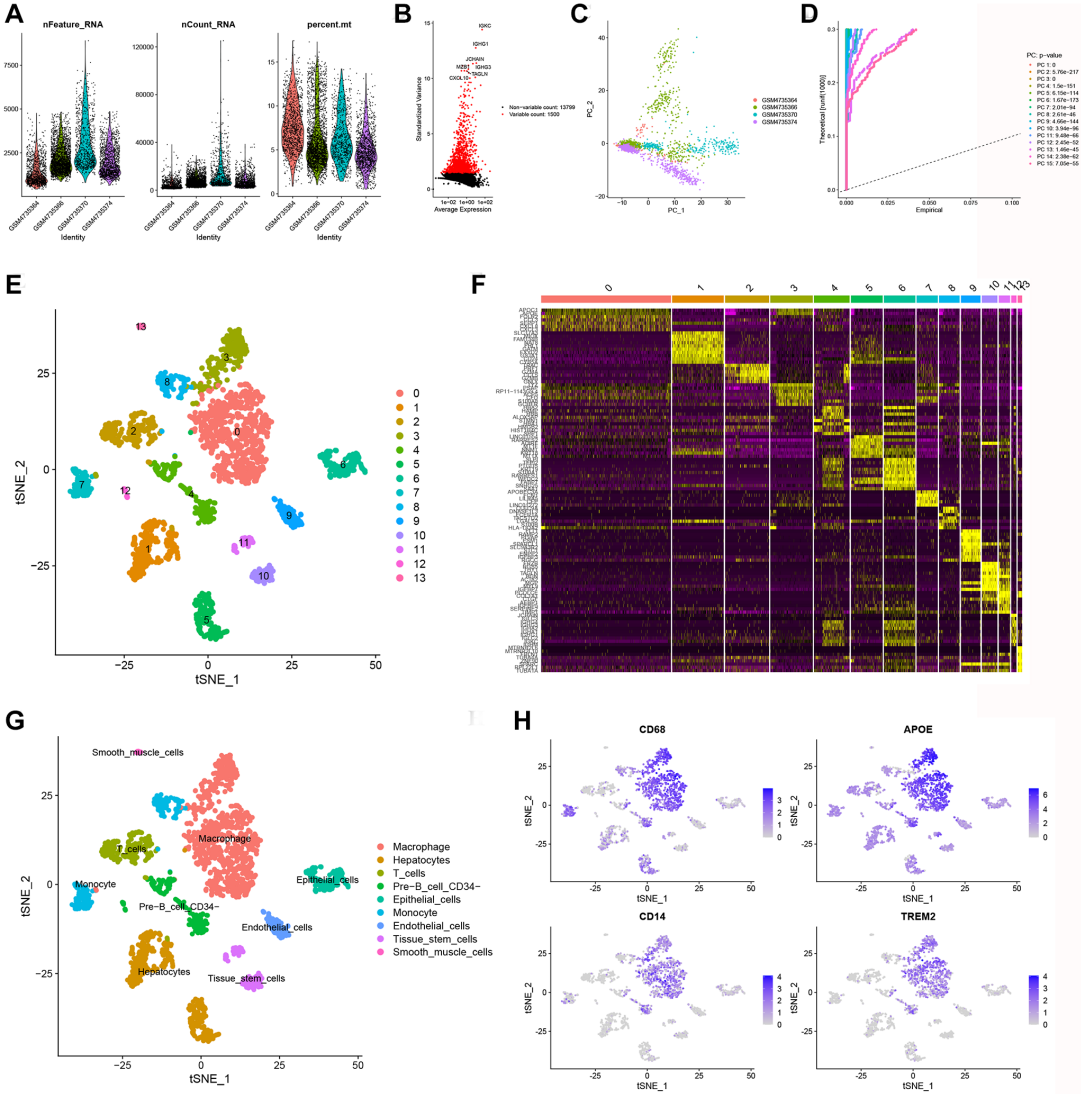
**Identification of MMGs via scRNA-seq analysis.** (**A**) Quality control of scRNA-seq data from four KIRC samples. (**B**) The variance plot shows that there are 13,799 genes in all cells, with the red dots representing the top 1,500 highly variable genes. (**C**) Dimensionality reduction was performed by PCA. (**D**) 15 PCs were identified based on a *P*-value < 0.05. (**E**) All cells were categorized into 14 clusters using the t-SNE algorithm. (**F**) The heatmap showed the top 10 most highly expressed genes in the 14 clusters. (**G**) Annotation of each cell cluster by marker genes. (**H**) Expression of known macrophage marker genes (CD68, APOE, CD14, and TREM2) in 14 cell clusters.

The scRNA-seq data were obtained from 5490 cells from four KIRC samples in the GEO database (GSE156632). After strict quality control filtering (removing low quality cells, mitochondria, etc.), a total of 2300 elevated expression cells were obtained (Figure 1A). After normalization of the data, the top 1500 of these highly expressed genes were selected (Figure 1B). PCA was conducted on four single-cell samples, resulting in a logical scattering and distribution of the individual samples (Figure 1C). To reduce dimensionality, we employed the PCA method and selected 15 principal components (PCs) with a *P*-value < 0.05 for subsequent analysis (Figure 1D). “Findnerghbors”, “FindClusters”, and “RunTSNE” functions were used to cluster the cells obtained above, and finally 14 clusters were obtained (Figure 1E). Next, using the “FindAllMark-ers” function, we screened a total of 1461 differentially expressed marker genes from 9 clusters. The top ten marker genes with the highest expression in each cluster were then clustered and visualized in a heatmap (Figure 1F). The t-SNE algorithm was performed to visualize the nine clusters. The “singleR” package, CellMarker database, and references were used for cell annotation (Figure 1G). And cluster 0 was defined as macrophage subpopulations, which were confirmed by marker genes (CD68, APOE, CD14, and TRME2) associated with macrophage based on CellMarker database (Figure 1H). Finally, we got 244 macrophage marker genes of KIRC (|logFC| > 1 and adjusted *P*-value < 0.05), which were listed in Supplementary Table 1.

### Construction and validation of a 5 macrophage marker genes-based prognostic model

**Figure 2:**
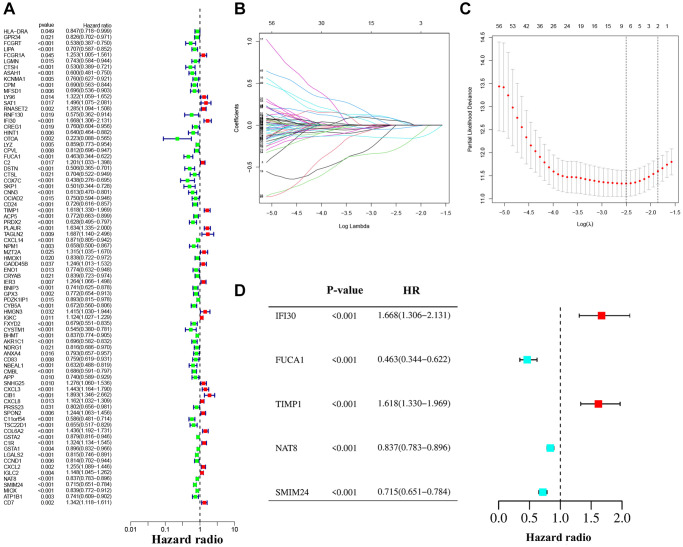
**The construction of the prognostic model.** (**A**) Univariate Cox regression analysis. (**B**, **C**) LASSO regression analysis. (**D**) Forest plot of multivariate Cox regression.

**Figure 3:**
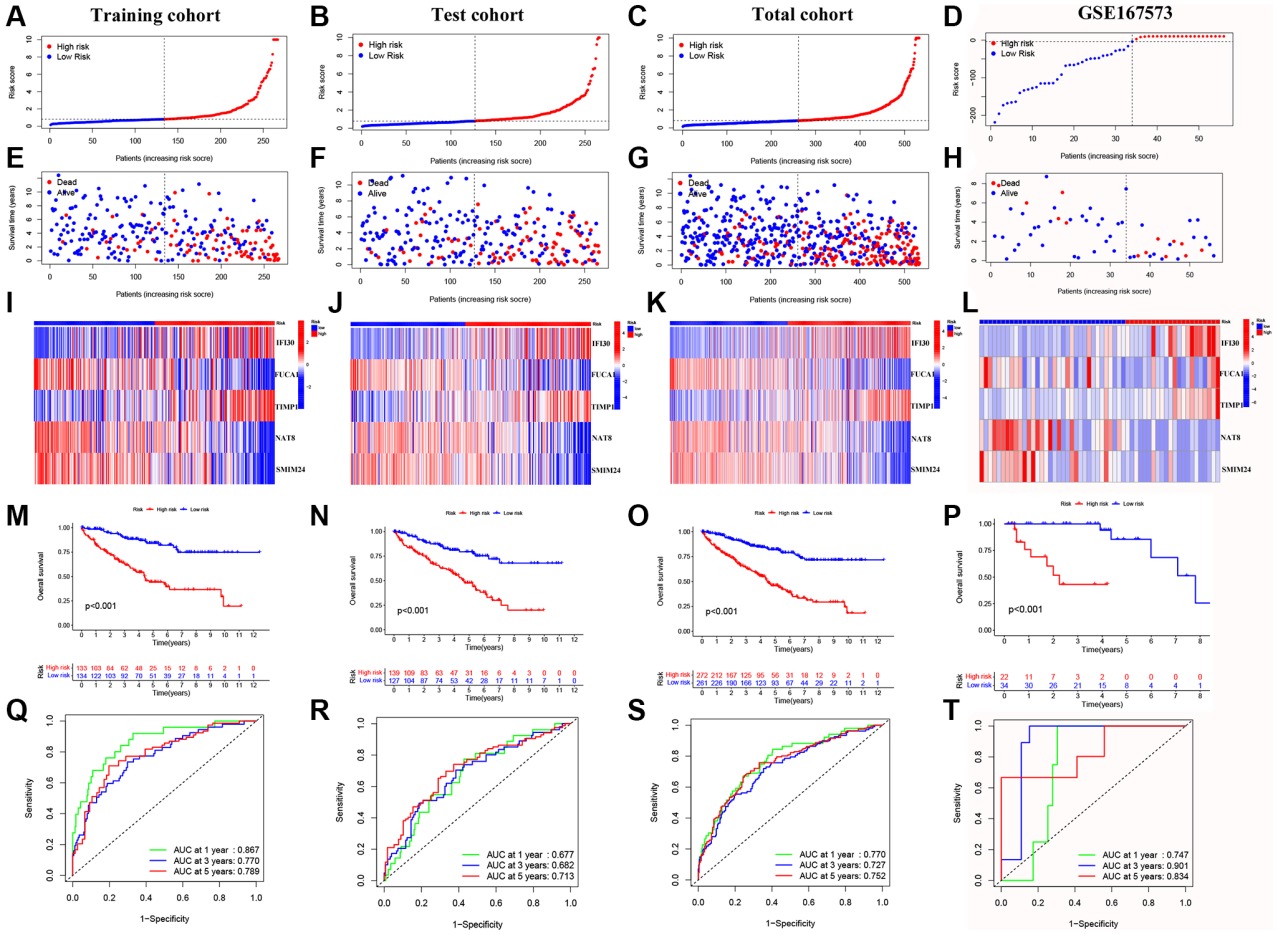
**Validation of the prognostic model.** (**A**–**D**) Distribution of risk scores for MMGs in TCGA-training, TCGA-test, TCGA-total and GEO cohorts, respective. (**E**–**H**) Scatter plot of the OS for each patient in the TCGA-training, TCGA-test, TCGA-total, and GEO cohorts, respectively. (**I**–**L**) Heatmaps of the risk cohort and the five MMGs in TCGA-training, TCGA-test, TCGA-total, and GEO cohorts, respectively. (**M**–**P**) The Kaplan-Meier curves in TCGA-training, TCGA-test, TCGA-total, and GEO cohorts, respectively. (**Q**–**T**) The AUC at 1-, 3-, and 5-year for the prognostic models in TCGA-training, TCGA-test, TCGA-total, and GEO cohorts, respectively.

**Figure 4:**
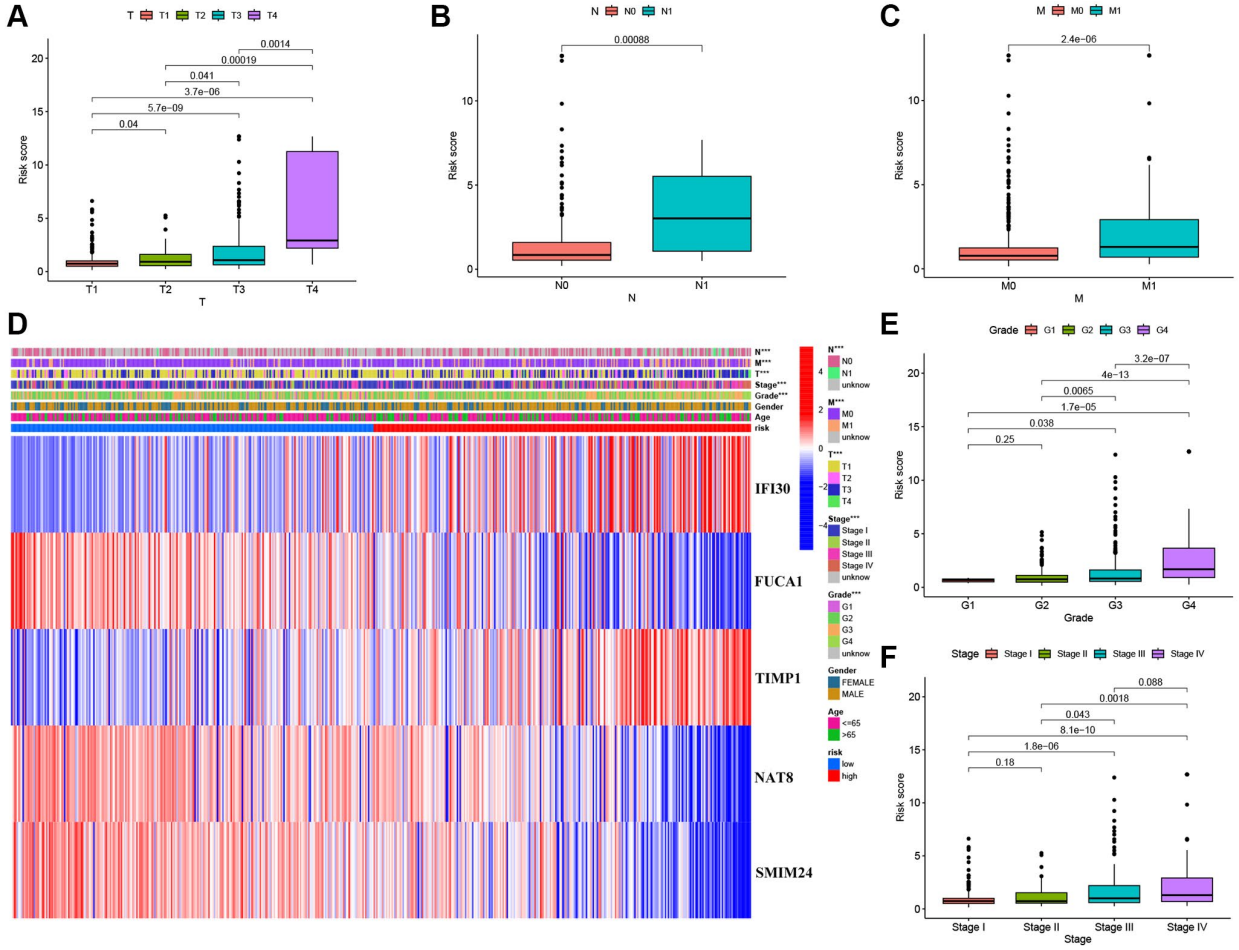
**Correlation between risk scores and clinical characteristics.** (**A**–**C**) Boxplot of risk scores based on MMGs signature for KIRC patients with different T status, N status, and M status. (**D**) Heatmap showed the relationship between clinical characteristics and expression of MMGs in the two risk groups. (**E**) Boxplot of risk scores based on MMGs signature for KIRC patients with different pathological grades. (**F**) Boxplot of risk scores based on MMGs signature for KIRC patients with different tumor stages.

**Figure 5:**
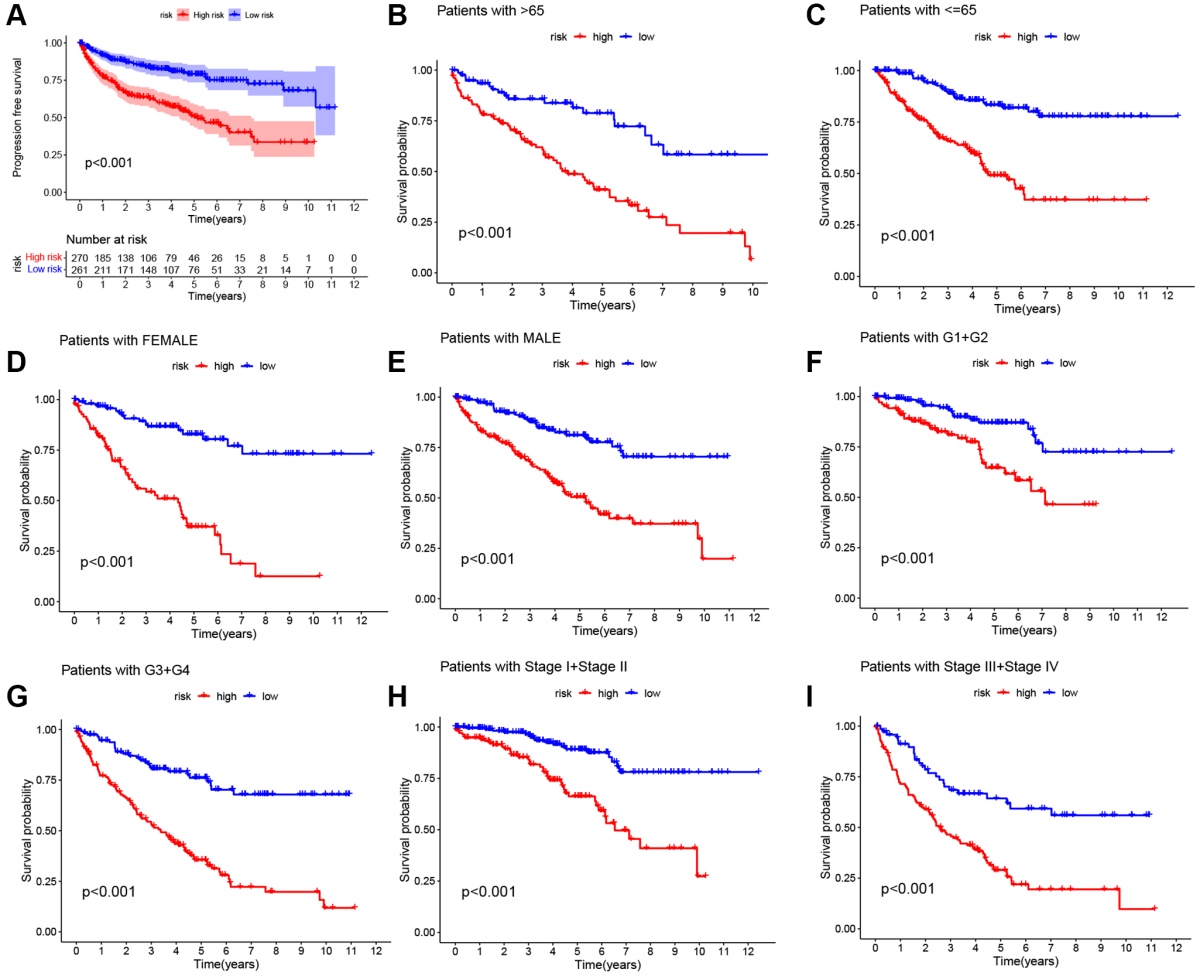
**Kaplan-Meier survival curves for two risk cohorts with different clinical characteristics.** (**A**) Progression free survival. (**B**, **C**) Age. (**D**, **E**) Sex. (**F**, **G**) Grade. (**H**, **I**) Stage.

We performed univariate Cox regression analysis on the TCGA-training set to screen for 81 macrophage marker genes that are associated with prognosis (Figure 2A, Supplementary Table 2). After LASSO and multivariate regression analysis, five genes were finally obtained (IFI30, FUCA1, TIMP1, NAT8, and SMIM24) (Figure 2B–2D). Then, based on correlation coefficients, a MMG risk was established: MMGrisk = (0.327 × IFI30 expression) + (−0.494 × FUCA1 expression) + (0.267 × TIMP1 expression) + (−0.108 × NAT8 expression) + (−0.145 × SMIM24 expression). KIRC patients were grouped into H-R and L-R cohorts based on median risk scores (Figure 3A–3D). The results suggest that as the risk score increases, the number of deaths increases and the survival time decreases (Figure 3E–3H), indicating a worse prognosis for the H-R group, which is also confirmed by the survival analysis results (*P* < 0.001). The expression of each MMG in the risk model was presented as a heatmap (Figure 3I–3L). The AUC for the 1-, 3-, and 5-year predictions in the TCGA-training cohort were 0.867, 0.770, and 0.789, respectively (Figure 3Q). The results of survival analysis showed poor prognosis in H-R cohort (Figure 3M). We used internal validation (TCGA test cohort) and external validation (GSE167573) to further evaluate the predictive power of the prognostic model. The results of TCGA-test and TCGA-total cohorts showed better OS in L-R cohort (*P* < 0.001) (Figure 3N, 3O), and the AUC in 1-, 3-, and 5-year were 0.677, 0.682 and 0.713, respectively (Figure 3R). In TCGA-total cohort, the AUC in 1-, 3-, and 5-year were 0.770, 0.727 and 0.752, respectively (Figure 3S). The results from the GEO cohort demonstrated that the OS of the L-R cohort was significantly better than that of the H-R cohort (*P* < 0.001) (Figure 3P). Additionally, the AUC for 1-, 3-, and 5-year predictions were 0.747, 0.901, and 0.834, respectively (Figure 3T). Moreover, in the KIRC cohort from the TCGA database, clinical characteristics were correlated with risk scores and showed as a heatmap (Figure 4D). Furthermore, correlation analysis showed a significant difference (*P* < 0.05) between risk score and T status (T1, T2, T3, and T4) (Figure 4A), N status (N0 and N1) (Figure 4B), M status (M0 and M1) (Figure 4C), pathologic grade (G1, G2, G3, and G4) (Figure 4E), and stage (stage I, stage II, stage III, and stage IV) (Figure 4F). Meanwhile, Kaplan–Meier survival curves were drafted between the two risk groups, and we found that the OS and progression-free survival (PFS) were substantially shorter in the H-R cohort (*P* < 0.05) (Figure 5A). The OS of the subgroups depending on clinical characteristics like age (Figure 5B, 5C), sex (Figure 5D, 5E), grade (Figure 5F, 5G) and stage (Figure 5H, 5I) revealed that the H-R cohort showed a poor prognosis compared to the L-R cohort in every subgroup. This result suggests that the model can better predict the prognosis of KIRC patients with different clinical variables.

### Nomogram construction and evaluation of predictive effectiveness

**Figure 6:**
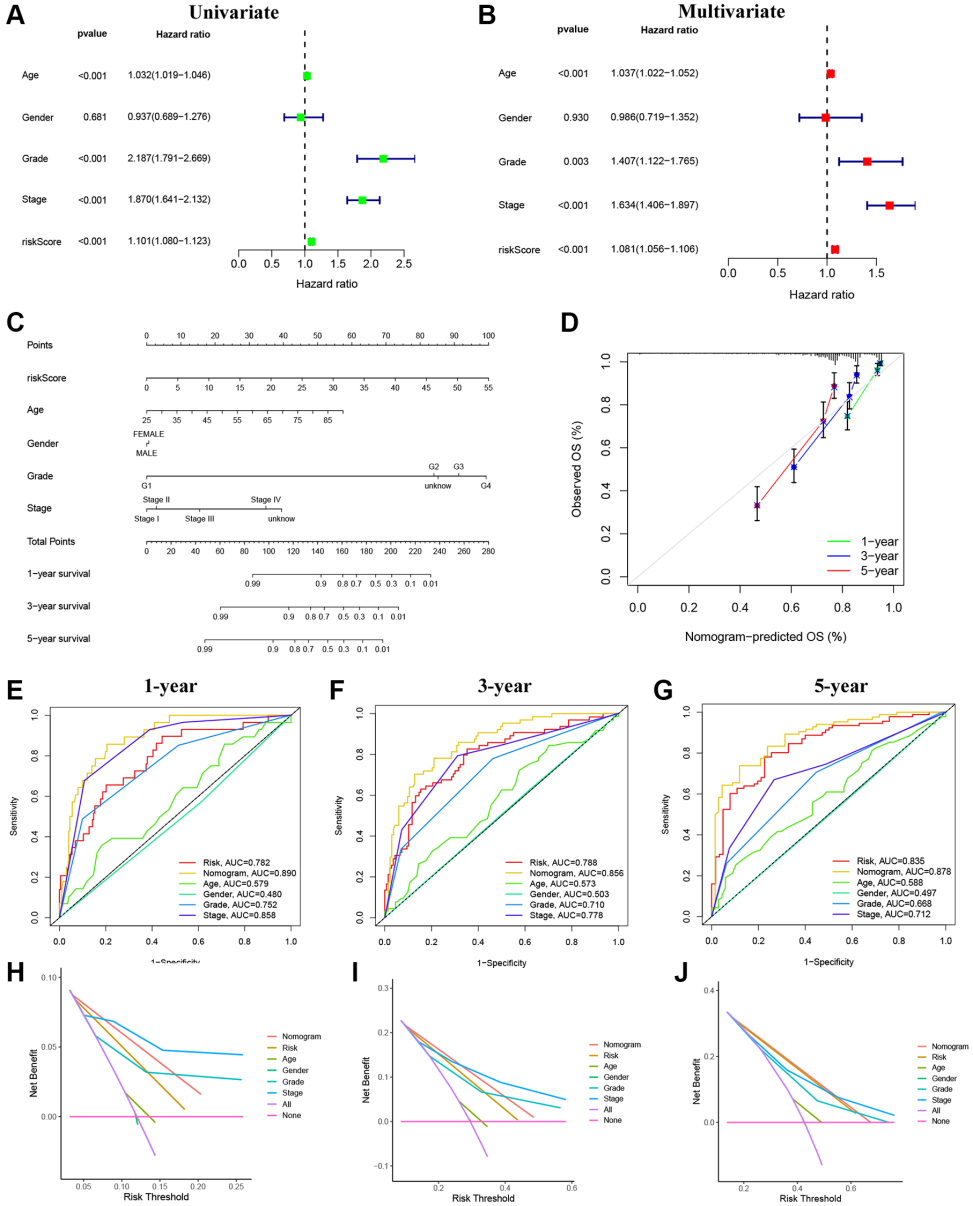
**Establishment and evaluation of the nomogram.** (**A**) Univariate Cox regression analyses of clinical characteristics and risk scores. (**B**) Multivariate Cox regression analyses of clinical characteristics and risk scores. (**C**) The construction of the nomogram. (**D**) Calibration curve for assessing the agreement at 1-, 3-, and 5-year OS. (**E**–**G**) The AUC of the nomograms compared for 1-, 3-, and 5-year OS, respectively. (**H**–**J**) The DCA curves of the nomograms compared for 1-, 3-, and 5-year OS, respectively.

Through univariate and multivariate regression analyses, we determined that age, grade, stage, and the risk score were independent prognostic factors influencing the outcomes of KIRC patients (Figure 6A, 6B). By integrating clinical characteristics and risk score, we developed a nomogram to predict the 1-, 3-, and 5-year OS of patients with KIRC (Figure 6C). The calibration curve demonstrates a strong agreement between the observed the and predicted values (Figure 6D). Moreover, the AUCs indicated the high accuracy of the nomogram in predicting 1-, 3-, and 5-year prognosis (Figure 6E–6G [13]). By plotting DCA curves, we found that this nomogram provided an excellent prediction of OS for patients with RCC for 1-, 3-, and 5-year (Figure 6H–6J).

### GO, KEGG and gene set enrichment analysis

**Figure 7:**
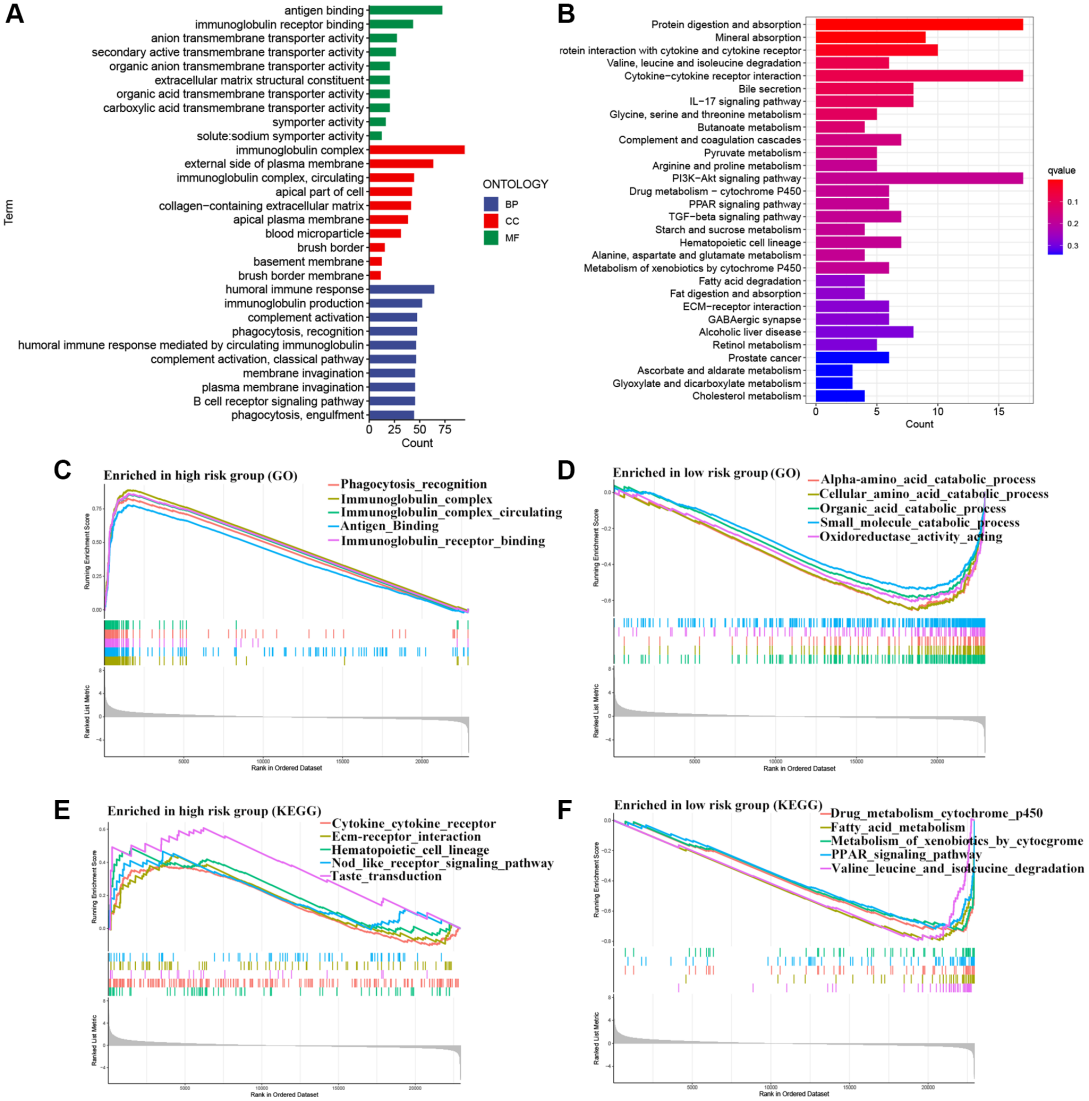
**Functional enrichment analysis.** (**A**) GO analysis. (**B**) KEGG analysis. (**C**–**F**) Gene set enrichment analysis.

Gene Ontology (GO) enrichment analysis was conducted, revealing that the biological processes (BP) primarily involved antigen binding and immunoglobulin receptor binding. In terms of cellular component (CC), the immunoglobulin complex, presynapse, and external side of the plasma membrane were the most prominently represented. In molecular function (MF), humoral immune response, and immunoglobulin production were the predominant categories (Figure 7A). KEGG analysis resulted mainly in protein digestion and absorption, cytokine-cytokine receptor interaction, and PI3K-AKT signaling pathway (Figure 7B). Furthermore, gene set enrichment analysis (GSEA) was conducted, and we found that the H-R group was mainly enriched in pathways related to immune function, such as immunoglobulin complex and immunoglobulin receptor binding (Figure 7C–7F). Next, we further performed an immune microenvironment analysis to compare the differences between the two risk groups.

### Analysis of tumor immune landscape in high- and low-risk groups

**Figure 8:**
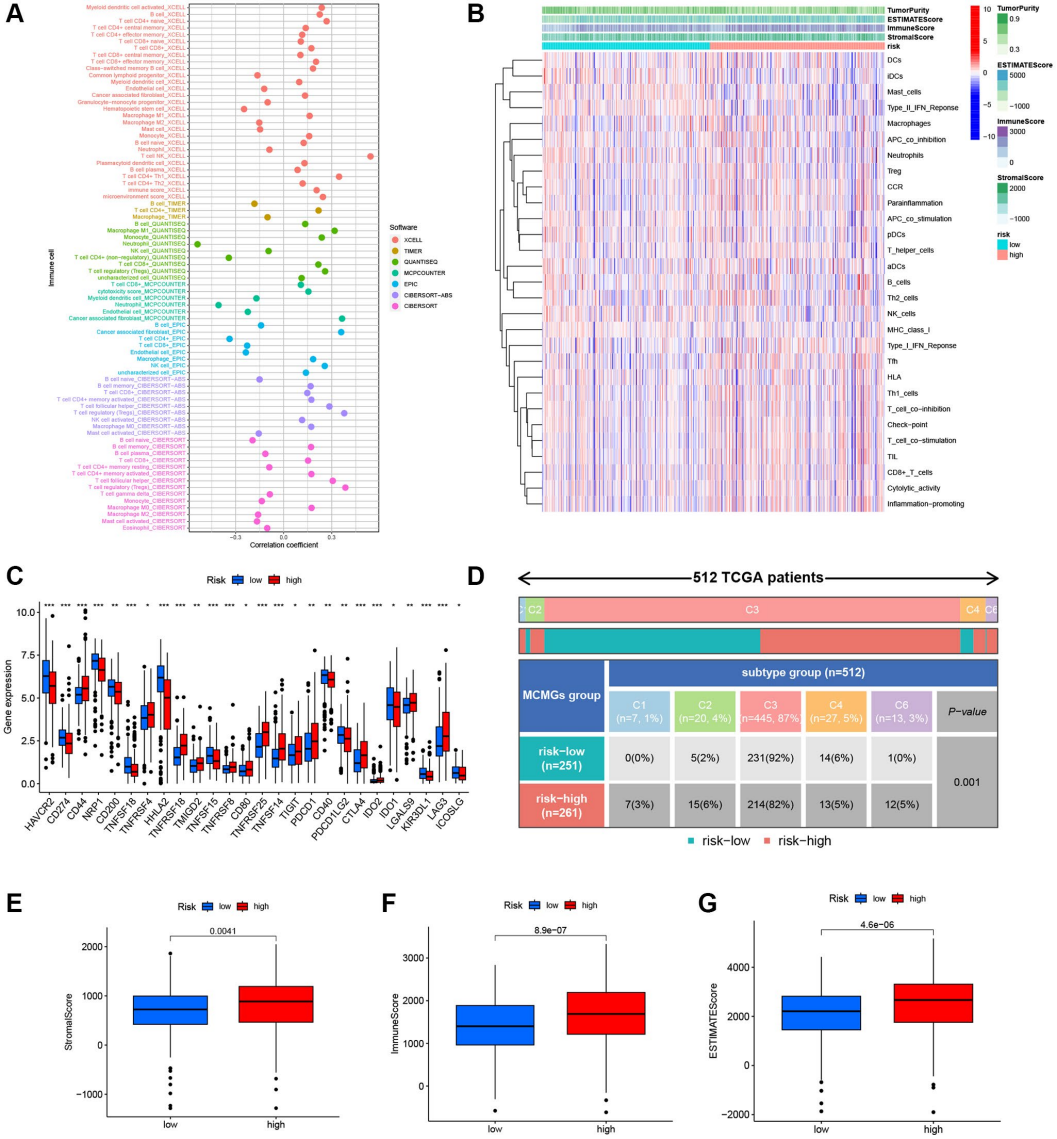
**Characteristics of the immune microenvironment of the tumor.** (**A**) Immune cell bubble of different risk cohorts. (**B**) Heatmap showed the relationship between MMGrisk and immune-related functions. (**C**) The expression levels of the immune checkpoint genes between the two risk groups. (**D**) Analysis of immune subtypes in the two risk cohorts. (**E**–**G**) Differences expression levels of stromal, immune, and ESTIMATE scores between the two risk cohorts.

We found that the H-R group was positively correlated with the infiltration of several crucial immune cells (Figure 8A), such as NK cells (R = 0.54), cancer associated fibroblast (R = 0.36), regulatory T cells (R = 0.39), and DC cells, and negatively correlated with neutrophil infiltration (R = −0.54). The details of the infiltrated immune cells are presented in Supplementary Table 3. Next, we illustrate the relationship between risk score and immune-related function in KIRC. The result showed numerous crucial immune functions were dramatically different in the risk scores, such as Type II IFN response, Macrophage, APC co-inhibition, HLA, T cell co-inhibition, and Inflammation promoting (Figure 8B). Compared to the L-R group, the H-R group exhibited higher expression of most immune checkpoint genes, including CD44, TNFRSF4, TNFRSF18, TMIGD2, TNFRSF8, CD80, TNFRSF25, TNFSF14, TIGIT, PDCD1, CTLA4, LGALS9, and LAG3 (Figure 8C). In addition, immunosubtype analysis showed that C3 subtype was predominant in the two risk cohorts (92% vs. 82%), the proportion of C1, C2, and C6 in the H-R group was higher than that in the L-R group (Figure 8D), and the difference was statistically significant (*P* < 0.05). Finally, as for the TME score, stromal score (*P* = 0.0041), immune score (*P* = 8.9e-07), and estimate score (*P* = 4.6e-06) were higher in H-R patients of KIRC (Figure 8E–8G).

### Tumor mutation and drug sensitivity analysis

**Figure 9:**
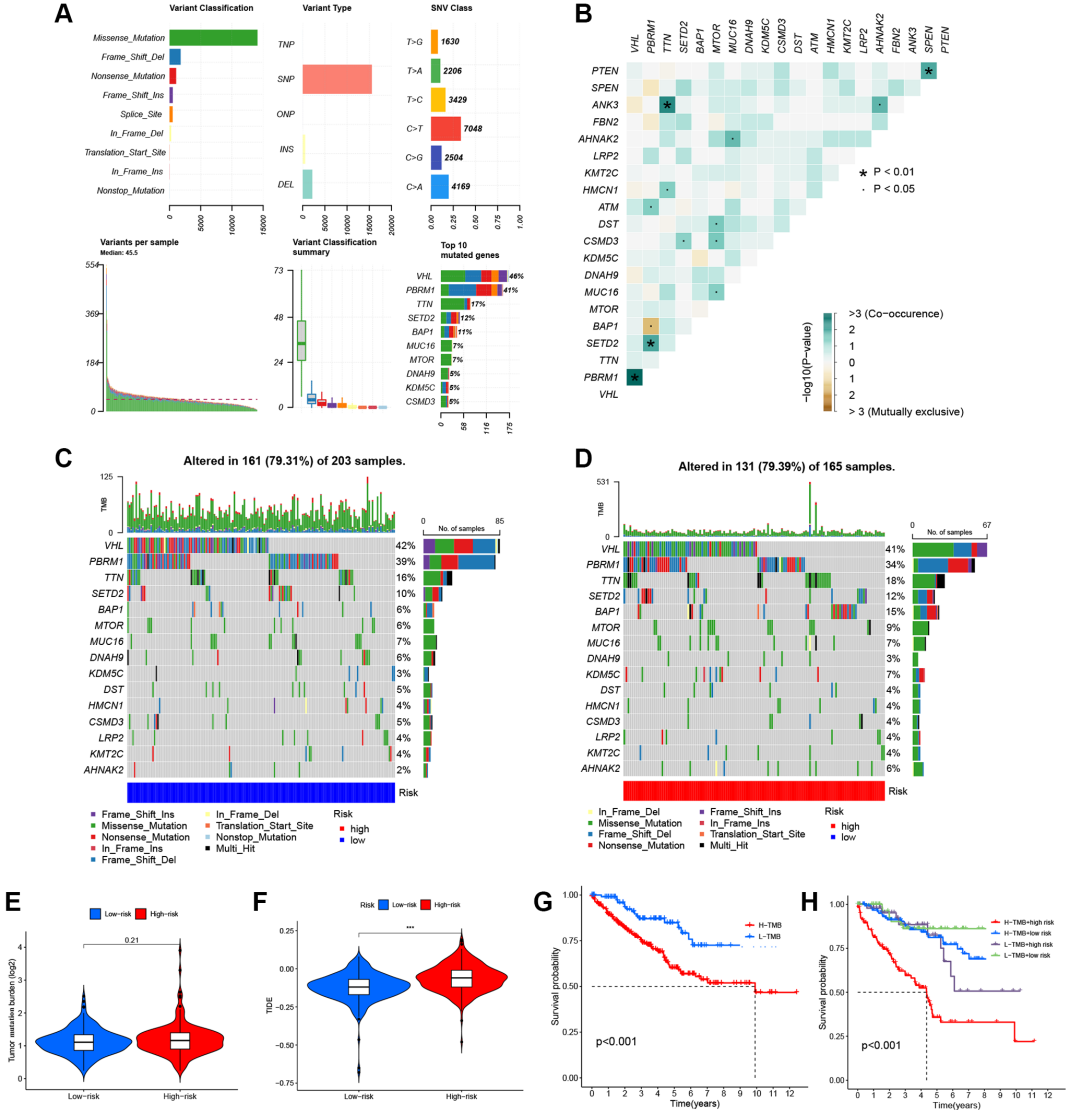
**Somatic mutations of the KIRC.** (**A**) The overall mutation profile of KIRC. (**B**) Interaction effect of gene mutation differentially in the two risk groups. (**C**) The mutation frequency of genes in the L-R group. (**D**) The mutation frequency of genes in the H-R group. (**E**) Differential expression levels of TMB between the two risk groups. (**F**) Differential expression levels of TIDE between the two risk groups. (**G**) The Kaplan-Meier curves for the low-TMB and high-TMB groups. (**H**) The Kaplan-Meier analysis curves for the patients stratified by MMGrisk and TMB.

**Figure 10:**
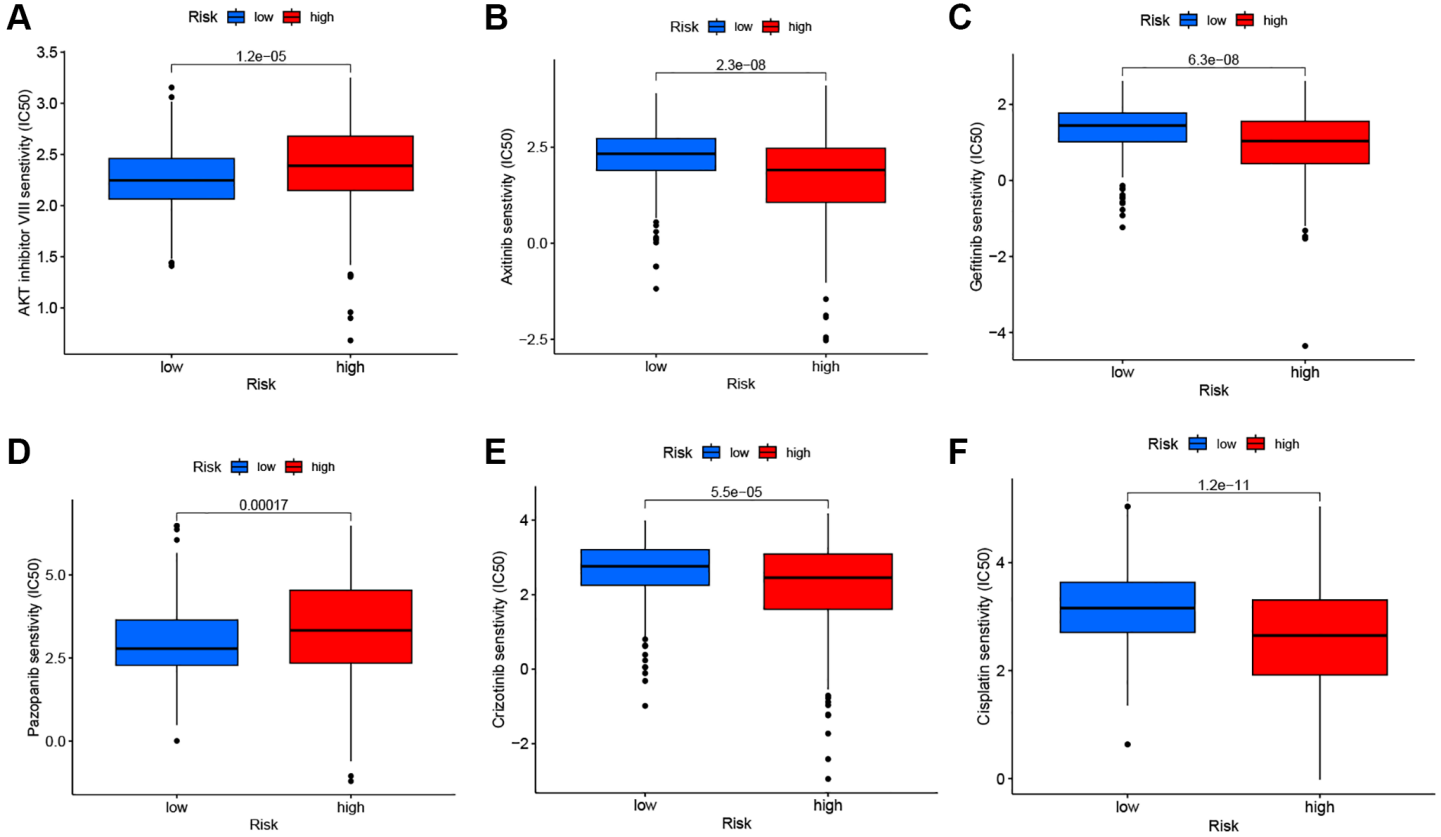
Drug sensitivity analysis. The comparisons in IC50 of AKT inhibitor (**A**), Axitinib (**B**), Gefitinib (**C**), Pazopanib (**D**), Crizotinib (**E**), and Cisplatin (**F**) between the two risk groups.

Figure 9A shows the overall mutation profile of KIRC. The interaction of genetic mutations is illustrated in Figure 9B. By analyzing the somatic mutation profiles of different risk groups from TCGA database, the top 15 most highly mutated genes were VHL, PBRM1, TTN, SETD2, BAP1, MTOR, MUC16, DNAH9, KDM5C, DST, HMCN1, CSMD3, LRP2, KMT2C, and AHNAK2 (Figure 9C, 9D). However, there was no significant difference in TMB between the two risk groups (*P* = 0.21) (Figure 9E). The OS was notably increased in the high TMB cohort, suggesting that the H-R cohort with low TMB had the worst prognosis (Figure 9G, 9H). There are two primary mechanisms by which tumor cells evade the immune system: the induction of T-cell dysfunction in tumors with high levels of cytotoxic T lymphocyte (CTL) infiltration, and the prevention of T-cell infiltration in tumors with low CTL levels. The TIDE can effectively model both mechanisms in order to predict the sensitivity to immunotherapy [14]. In comparison with the L-R cohort, the TIDE scores of the H-R cohort were substantially higher (Figure 9F), further indicating that L-R patients benefit more from the immunotherapy. We subsequently utilized the “pRRophetic” package to delve deeper into the disparities in IC50 levels among various drugs within the L-R and H-R groups. The results showed that patients in the L-R group had lower IC50 scores for cancer drugs including AKT inhibitors and Pazopanib. On the contrary, patients in the H-R cohort had lower IC50 for the anti-cancer drugs including Axitinib, Gefitinib, Crizotinib, and Lisitinib (Figure 10A–10F). The results of the correlation between drug IC50 and risk score are shown in Supplementary Figure 1. These results suggest that this predictive model can be used as a predictor of the efficacy of anti-tumor drugs and as a reference for the precise treatment of kidney cancer patients.

### Validation of signature gene expression biological function analysis

**Figure 11:**
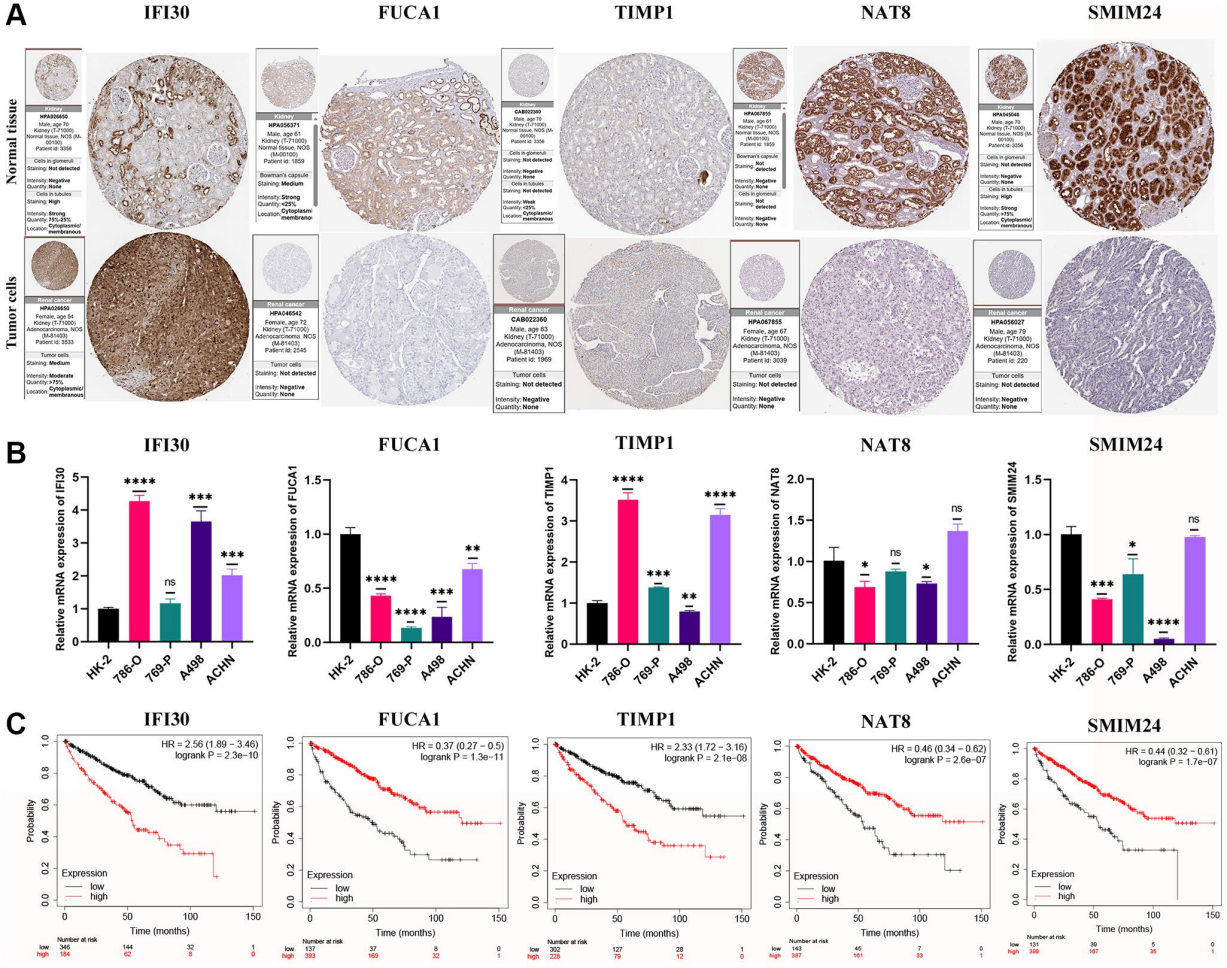
**Validation of expression and survival of model genes.** (**A**) The protein expression profiles of the key genes in the Human Protein Atlas (HPA) database. (**B**) The mRNA expression level of the key genes in a renal tubular epithelial cell line (HK-2) and KIRC cell lines (786-O, 769-P, A498, and ACHN). (**C**) Survival analysis for the key genes. Error bars are mean ± SD, ^*^P < 0.05, ^**^P < 0.01, ^***^P < 0.001.

**Figure 12:**
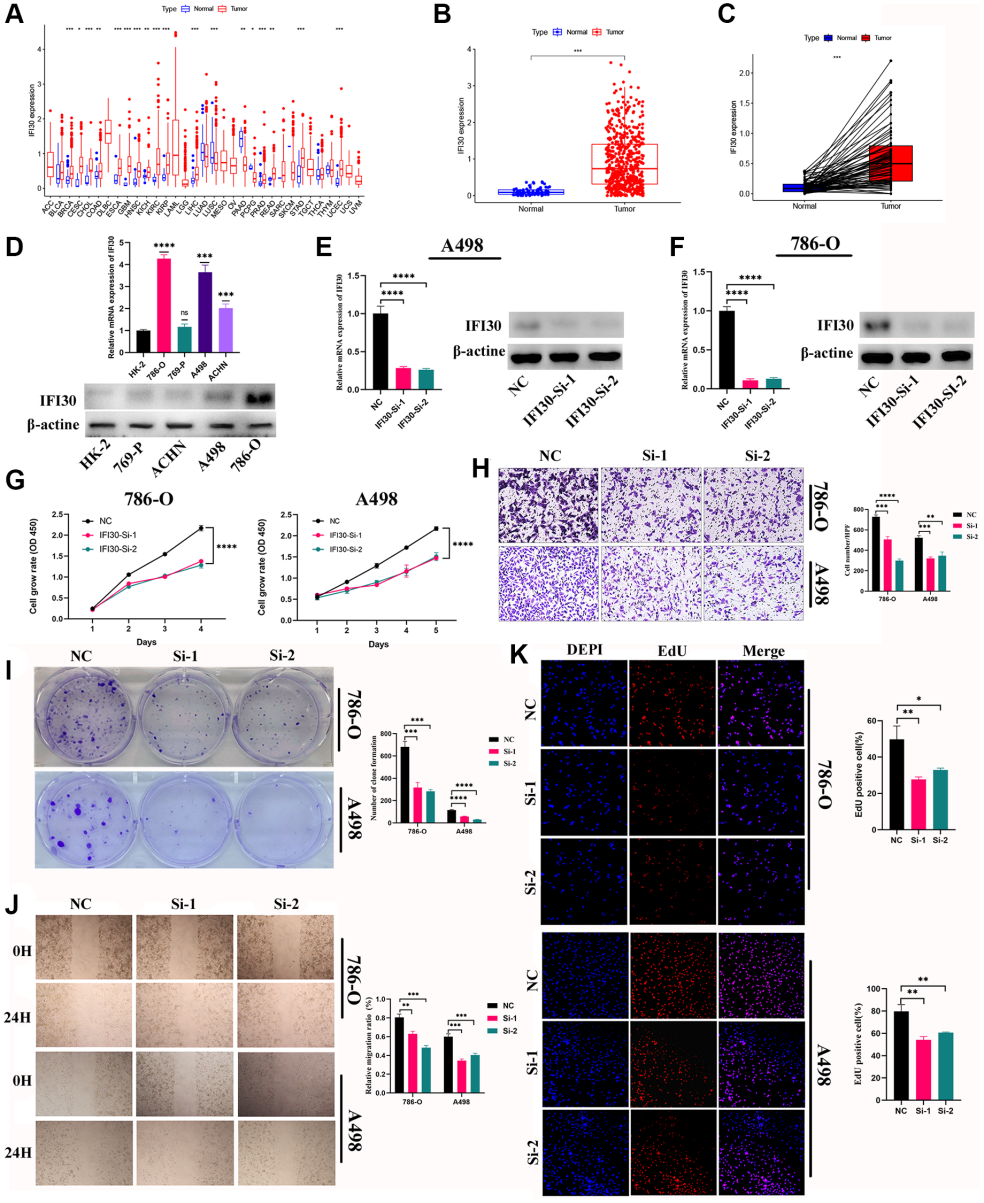
**The role of IFI30 in KIRC.** (**A**) Pan-cancer analysis of IFI30 expression in human tumors. (**B**) Expression of IFI30 in KIRC and para-cancerous tissues in TCGA database. (**C**) Expression of IFI30 in KIRC and normal tissues in TCGA database. (**D**) IFI30 was highly expressed in KIRC cell lines compared to normal renal tubular epithelial cell line (HK-2). (**E**, **F**) RT-qPCR and Western blot were used to verify the interference efficiency of IFI30 in A498 and 786-O cells. (**G**) CCK8 assay. (**H**) Transwell assay. (**I**) Colony formation assay. (**J**) Wound healing assay. (**K**) EdU staining assay. Error bars are mean ± SD, ^*^P < 0.05, ^**^P < 0.01, ^***^P < 0.001.

The expression levels of marker genes (IFI30, FUCA1, TIMP1, NAT8, and SMIM24) in our prognostic model were validated in mRNA and protein levels. We found that the expression of IFI30 and TIMP1 is higher in the RCC cell lines than that in normal renal tubular epithelial cells, and is associated with a poorer prognosis. The expression of FUCA1, NAT8 and SMIM24 in renal carcinoma cell lines is lower than those in renal tubular epithelial cells and suggests a better prognosis (Figure 11). Then, we selected the IFI30 with the highest hazard ratio (HR) for additional study. Pan-cancer analysis revealed that IFI30 is highly expressed in most human tumors compared with normal tissue (Figure 12A). As for KIRC, the expression of IFI30 in tumor samples is significantly higher than that in non-tumor samples, both in paired and non-paired samples (Figure 12B, 12C). The above analysis was further corroborated by the significantly higher expression of IFI30 in KIRC cell lines than in normal renal tubular epithelial cells (Figure 12D). Next, IFI30 was knocked down in RCC cell lines for subsequent functional experiments in the A498 and 786-O cells. Initially, we evaluated the knockdown efficiency of IFI30 at the RNA level and the protein level, respectively (Figure 12E, 12F), the original Western blot and gels were shown in Supplementary Figure 2. CCK8 and EdU assays showed that knockdown of IFI30 significantly inhibited the proliferation of A498 and 786-O cells (Figure 12G, 12K). In addition, results from colony formation assay showed that the knockdown of IFI30 resulted in a significant reduction in cell colonies compared with the controls (Figure 12I). This is corroborated by the results of wound healing assay, which show that after inhibition of IFI30 expression, the wound healing rate of cells is significantly decelerated (Figure 12J). Similarly, knockdown of IFI30 inhibited the migration of A498 and 786-O cells (Figure 12H). To verify the accuracy and reliability of the above results, experiments were performed in A498 and 786-O cells in a triplet manner. All data were expressed as the means ± SD of independent experiments. ^*^P < 0.05, ^**^P < 0.01, ^***^P < 0.001.

## DISCUSSION

In the TME, TAMs are the most abundant immune-associated stromal cells. On the one hand, macrophage phagocytosis results in the elimination of tumors, activation of inflammasomes, and presentation of antigens, thus triggering adaptive immune responses against the tumors [15]. On the other hand, macrophages may also contribute to tumor advancement, metastasis, and resistance to therapy [16]. While macrophages undoubtedly play a pivotal role in the TME and patient outcomes, the relationship between MMGs and outcomes as well as immune response in KIRC patients remains uncertain.

In this study, the prognostic signature constructed by integrating scRNA-seq and bulk RNA-seq data consists of five MMGs (IFI30, FUCA1, TIMP1, NAT8, and SMIM24). It has been reported that IFI30 can promote the EMT process in glioma cells by activating EGFR/AKT/GSK3β/β-catenin. In addition, IFI30 directly modulates drug resistance in glioma cells [17]. Fan et al. elaborated that IFI30 is highly expressed in breast cancer and is associated with poor prognosis, and that it can affect breast cancer cell proliferation by regulating autophagy [18]. Activation of the AKT signaling pathway is facilitated by TIMP1, which has been demonstrated to confer resistance to CisPt and Dox in TNBC patients. It is worth nothing that the formation of the TIMP1/CD63 complex plays a crucial role in this process [19]. Baudot et al. [20] reported that P53 can directly regulate FUCA1 and promote chemotherapy-induced cell death. Using *in vitro* and *in vivo* functional experiments, Xu et al. [21] showed that FUCA1 silencing inhibited glioma growth by enhancing autophagy and inhibiting macrophage infiltration. It has been reported that NAT8 is regulated by FDFT1 and promotes colon cancer cell proliferation *in vivo* and *in vitro*. You et al. reported that NAT8 is a CAF-related methylation driver gene that favors CAF infiltration in KIRC and is associated with sensitivity to immune checkpoint inhibitors, and that its methylation level is negatively correlated with the prognosis in KIRC patients [22]. Tong et al. screened out six DEGs (including NAT8) which downregulated in ccRCC patients with VHL non-mutation than with the mutation, and its decreased expression is associated with a better prognosis in ccRCC patients [23]. The role and mechanism of SMIM24 in tumors has not been reported. This study is the first to comprehensively assess the impact of MMGs in patients with KIRC in terms of clinical outcomes, immune profile, and drug sensitivity.

Our team conducted both internal validation (using the TCGA-test dataset) and external validation (utilizing the GEO cohort) of the prognostic signature we obtained earlier. The consistently positive results in the validation set demonstrate excellent reproducibility. Subsequently, we developed a nomogram to predict the OS of KIRC patients at 1-, 3-, and 5-year. Furthermore, GO enrichment analysis revealed that the MMGs in the signature are predominantly enriched in immune-related pathways, such as immunoglobulin receptor binding, immunoglobulin complex, and humoral immune response. KEGG analysis revealed enrichment of MMGs in protein digestion and absorption, cytokine-cytokine receptor interaction, and PI3K-AKT signaling pathway. These pathways have been reported to be associated with immunotherapy response and TME [24–26]. The results of the GSEA analysis further validate the confidence of the above-enriched signaling pathways.

TAMs play a role in promoting cancer cell growth and metastasis, as well as exerting immunosuppressive effects on adaptive immune cells within the TME. The study’s findings suggest that patients in the H-R group demonstrated significantly higher immune scores, stromal scores, and ESTIMATE scores (*P* < 0.05). In addition, our team detected widespread infiltration of immune cells and elevated expression of genes associated with immune checkpoints in the H-R group. These findings suggest that individuals within the H-R cohort have a less favorable prognosis compared to those in the L-R cohort. This is due to the fact that immune checkpoint genes, such as CD274, cause T cell dysfunction, hindering the ability of cytotoxic T cells to target tumor cells. As a result, this interference allows tumor cells to evade immune surveillance and promotes tumor progression [27, 28].

Gene mutations are key factors in tumor formation, and identifying specific gene mutations through second-generation sequencing has the potential to provide the basis for precision targeted therapies. Our team found that the most predominant mutations in KIRC patients were missense mutations, with C > T Single nucleotide polymorphism (SNPs) being the most common type. In these mutant genes, BAP1 mutation frequency in the H-R group was significantly higher than that in the L-R group (15% vs. 6%). Kaler et al. found that BAP1 deficiency leads to increased PROS1 expression in melanoma cells, polarizes macrophages toward the M2 state, activates the suppressive tumor immune microenvironment, and ultimately promotes immune escape [29]. The H-R group in this study had a higher TIDE score, which may be related to activation of the tumor-suppressing immune microenvironment due to polarization of M2 macrophages. In addition, we found that the H-R group with elevated TMB had the worst prognosis, whereas the L-R group with low TMB had the best prognosis.

To assess the predictive power of prognostic signatures in terms of sensitivity to common chemotherapeutic and anti-angiogenic agents, our team investigated AKT inhibitors, Axitinib, Gefitinib, Pazopanib, Cirzotinib, and Cisplatin. The findings suggest that higher risk scores are associated with an increased IC_50_ for both AKT inhibitors and Pazopanib. In turn, a higher risk score is associated with a reduced IC_50_ for Axitinib, Gefitinib, Cirzotinib, and Cisplatin. Moreover, the GSEA analysis results demonstrate that the model signatures exhibit significant enrichment in drug metabolism related pathways. This further confirms that signatures have the potential to influence drug sensitivity and provides a basis for selecting targeted drugs for patients with KIRC.

Finally, we performed a comprehensive pan-cancer analysis on IFI30, which was identified as having the highest HR value among the model genes. Our findings reveal elevated expression of IFI30 across multiple human tumors, associated with an unfavorable prognosis. We observed elevated levels of IFI30 in KIRC cells compared to normal cells. Subsequently, cell function experiments were performed using the A498 and 786-O cell lines. The results demonstrate a significant reduction in cell invasion, migration, and proliferation in KIRC cells following IFI30 knockout. Still, our study has some limitations. First, our study is based on the analysis and summarization of existing public databases, and a large number of real-world clinical samples for validation is still lacking. Second, further *in vivo* and *in vitro* experiments are needed to validate the mechanisms involved in this study. In the future, we will rely on our Tianjin Key Laboratory for Precision Medicine of Sex Hormones and Diseases to carry out our real-world clinical studies and verify in-depth molecular mechanisms.

In summary, our team developed a prognostic model, including five macrophage marker genes, to predict prognosis and response to drug therapy in patients with KIRC. In addition, the discovery of IFI30 as a potential new target suggests that it could be a valuable component in the development of personalized therapies for KIRC. This research holds promise for improving the management and outcomes of KIRC patients.

## MATERIALS AND METHODS

### Data source

Four scRNA-seq files (GSM4735364, GSM4735366, GSM4735370, GSM4735374) were acquired from the GEO database (http://www.ncbi.nlm.nih.gov/geo/ (accessed on 3, August 2023) accession number GSE156632 to acquire macrophage cell marker genes. Next, bulk RNA-seq data, matched clinical information annotations, and somatic mutation data were obtained from the TCGA database (https://portal.gdc.cancer.gov/ (accessed on 3, August 2023), including 72 normal kidney samples and 542 KIRC samples. A total of 533 KIRC samples were included in the analysis after excluding patients with tumors for which no survival data were available. Furthermore, an external validation cohort and the corresponding clinical information were downloaded from the GEO database (GSE167573).

### Single cell RNA-seq analysis

ScRNA-seq data analysis was done by using the “Seurat” package [30]. Cells with more than 5 percent of the mitochondrial genes were removed. In addition, cells with fewer than 50 mapped genes and clusters with less than 3 cell counts were eliminated. To perform dimensionality reduction using Principal Component Analysis (PCA), we used 1500 highly variable genes. The optimal clusters were then selected for visualization using Seurat’s Stochastic Neighbor Embedding (t-SNE) algorithm [31], “FindNeighbors”, and “FindClusters” (resolution = 0.5) function [32]. Next, the differences in gene expression between a specific cluster and all other clusters were compared using the “FindAllMarkers” function. And genes that exhibited a |log2FC| > 1 and adjusted *P*-value < 0.05 were considered as the marker gene. Finally, the “SingleR” package [33] was used to annotate the cells of different subpopulations.

### Construction and validation of the prognostic signature

Firstly, by using the R package “limma” [34], we integrated expression profile data and clinical data of 533 KIRC patients from the TCGA database, and then randomly divided into a training cohort (*n* = 267) and a test cohort (*n* = 266) in a 1:1 ratio through “cart” package. Secondly, we performed univariate Cox regression analysis in the training cohort to identify macrophage marker genes (MMGs) associated with OS. Third, LASSO and multivariate Cox regression were performed to select hub genes to construct the risk model. And MMG risk score calculating formula was:


$$MMGriskscore\sum_{i=1}^{n}coef(i)\times x(i)$$

Where *x* (*i*) = each MMG expression, and coef (*i*) = regression coefficient. The patients (training cohort, test cohort and total cohort) were divided into H-R and L-R cohorts based on the median value of the riskscore. The R package “survival” was applied to analyze the survival of the three data sets. Finally, “glmnet”, “caret”, “survminer”, “rms”, and “timeROC” packages were used to plot the ROC and calculate the AUC to evaluate the accuracy of the prognostic model. In addition, GSE167573 was used as an external validation dataset to evaluate the prognostic signature. And a KM survival analysis to validate the prognostic potential of risk genes based on the Kaplan-Meier plotter (http://kmplot.com (accessed on 5, August 2023) database.

### Expression of marker genes at the mRNA and protein level

We analyzed the expression of the above-obtained marker genes at the transcriptome and protein levels. For the mRNA level, we performed RT-qPCR validation on renal cancer cells (ACHN, 786-O, 769-P, and A498) and normal renal tubular epithelial cells (HK-2). For protein level, we obtained immunohistochemical (IHC) staining images of the marker genes from the Human Protein Atlas database (HPA; https://www.proteinatlas.org/ (accessed on 10, August 2023) [35].

### Clinical characteristic, nomogram and decision curve analysis

Univariate and multivariate Cox regression analysis were used to assess whether risk score and clinical characteristics (grade, sex, stage, and age) were independent factors that affect prognosis of KIRC. Next, a nomogram was constructed to predict the OS of KIRC individuals at 1-, 3-, and 5-year by the “rms” package [36, 37]. The receiver operating characteristic (ROC) curves and calibration curve were used to assess the nomogram’s predictive power accuracy [38]. Based on the “ggDCA” package, decision curve analysis (DCA) was performed to evaluate the net clinical benefit of the signature and clinical factors on KIRC patient survival outcomes.

### Functional enrichment analysis

To clarify the function of core genes and underlying biological functions and mechanisms, we conducted KEGG and GO analysis by using “ClusterProfiler”, “org.Hs.eg.db” [39], “ggplot2”, “enrichplot” packages. We performed GSEA on the two risk groups identified in the TCGA cohort using the Molecular Signature Database (MSigDB) (specifically, c2.cp. KEGG gene set and c2.cp. GO gene set). We then visualized the top 5 pathways with a significance level of *P* < 0.05 for each human gene ensemble.

### Tumor immune microenvironment and immunotherapy

Seven algorithms (XCELL, TIMER, QUANTISEQ, MCPCOUNTER, EPIC, CIBER-SORT-ABS, CIBERSORT) [40–46] were used to evaluate immune infiltration in KIRC patients. These data were used to analyze the infiltrating abundance of immune cells in the tumor microenvironment. Next, we used the “estimate” package [47] to determine the relative expression abundance of stromal cells, immune cells, and tumor cells in two risk groups. A higher score indicates a greater presence of each component in the tumor microenvironment. We compared the expression levels of established immune checkpoint genes (ICGs) between the two risk groups using the “limma”, “reshape2”, “ggplot2”, and “ggpubr” packages. Immune subtype (C1: Wound Healing, C2: IFN-gamma Dominant, C3: Inflammatory, C4: Lymphocyte Depleted, C5: Immunologically Quiet, C6: TGF-beta Dominant) [48] distribution difference was further compared in two risk groups by “RcolorBrewer” package.

### TMB, TIDE and mutation analysis

We obtained somatic mutation data for KIRC patients from the TCGA database and processed them using the Perl language. We utilized the “maftools” package to identify the TMB score and survival data for both risk cohorts [49], and top 15 genes with the highest mutation frequency in both groups. KIRC patients were divided into high-TMB and low-TMB groups based on their median TMB score. K-M survival curve was performed to analyze the overall survival of the two risk groups by using “survival” and “survminer” packages. Finally, the TIDE score files were downloaded from the TIDE website (http://tide.dfci.harvard.edu/ (accessed on 15, August 2023) [50].

### Drug sensitivity analysis

The Genomics of Drug Sensitivity in Cancer (GDSC) database was utilized to evaluate the sensitivity of KIRC patients to drug treatment. This assessment was performed using the “pRRophetic” package [51], where we assessed treatment response based on 50% maximum inhibitory concentration (IC_50_).

### Cell lines culture and RNA interference

Normal human renal tubular epithelial immortalized cells (HK-2) and KIRC cells (ACHN, 786-O, 769-P, and A498) were obtained from Tianjin Institute of Urology, The Second Hospital of Tianjin Medical University. HK-2 and ACHN cells were cultured in DMEM medium (MA0212, Meilunbio, Dalian, China). 786-O, 769-P, and A498 cells were cultured in RPMI-1640 medium (FI201-01, Transgen, Beijing, China). All the medium were supplemented with 10% fetal bovine serum (FS401-02, Transgene, Beijing, China) and 1% streptomycin/penicillin (S110JV, BasalMedia, Shanghai, China), and all the cells were incubated in a humidified atmosphere with 5% CO_2_ at 37°C. The short interfering RNA (siRNA) probe targeting IFI30 was designed and synthesized by GenePharma (Suzhou, China). All transfections were conducted using RFect Transfection Reagent (11012, Baidai, Changzhou, China). The siRNA sequences for IFI30 can be found in Table 1.

**Table 1:** Primer sequence of genes.

**Gene**		**Sequence (5′–3′)**
GAPDH	F	GGGGAGCCAAAAGGGTCATCATCT
	R	GACGCCTGCTTCACCACCTTCTTG
IFI30	F	GCGTTAGACTTCTTTGGGAATGGGC
	R	ACCGCACAGTGCTTCATAGTAGAGG
IFI30-Si-1	Sense	CCAGCCACCACACGAGUAUTT
	Antisense	AUACUCGUGUGGUGGCUGGTT
IFI30-Si-2	Sense	GCAAGCGUUAGACUUCUUUTT
	Antisense	AAAGAAGUCUAACGCUUGCTT
FUCA1	F	AAGGCTTCTTGCTGTTGGGAAATGG
	R	GCATAAACAGCCGATCCCTTTGAGG
TIMP1	F	TTCTGGCATCCTGTTGTTGCTGTGG
	R	GTGGTCTGGTTGACTTCTGGTGTCC
NAT8	F	GGGATAGCAAAAGCCCTGGT
	R	TCTTGAAGCCCATGCTCTGG
SMIM24	F	TCATCGTCTATTTGGTCTTGCTGGC
	R	CTCCATTCTGAACGTGGTCTCCTCC

### Real-time quantitative polymerase chain reaction (RT-qPCR)

Trizol reagent was used to extract RNA from the cell line. cDNA was generated using the Reverse Transcription Kit (BL696A, Biosharp, Beijing, China) according to the manufacturer’s protocol. Furthermore, TOROGreen qPCR Master Mix (AQ131-02, Transgene, Beijing, China) was applied for qRT-PCR. The relative RNA expression levels were quantified using the 2^−ΔΔCt^ method, with GAPDH serving as the internal control. The sequence of primers can be found in Table 1.

### Western blot

RIPA buffer (AR0103-100, BOSTER, Wuhan, China), PMSF (P0110, Solarbo, Beijing, China) and protease inhibitor (P6730, Solarbo, Beijing, China) (100:1:1) were used to obtain total protein and we used the BCA method to determine the total protein concentration. Proteins were separated using a 10% SDS/PAGE gel and subsequently transferred onto a PVDF membrane (ISEQ00010, Millipore, USA). The membrane was then blocked with 5% skimmed milk for membrane closure, followed by incubation with the primary antibody overnight at 4°C. After binding of the primary antibody, the membrane was incubated for 1 hour at room temperature with a secondary antibody tagged with horseradish peroxidase. The final exposure was detected with the ECL luminescent reagent. The antibodies used in this study were as follows: IFI30 (11597-1-AP, Proteintech, Wuhan, China), β-Actine (66009-1-Ig, Proteintech, Wuhan, China).

### CCK8 and colony formation analysis

KIRC cells from the logarithmic growth phase were taken, and both control and treated cells were digested and resuspended into single cell suspensions and counted. Equal amounts of cell suspension (1000 cells/well) were added to 96-well plates with 3 subwells per group and placed in a cell incubator (37°C, 5% CO_2_). The plates were incubated for 24, 48, 72, and 96 hours in a cell-in-incubator. Add 200 ul of assay solution (180 ul complete medium + 20 ul CCK8 reagent) to each well and put the plates back into the incubator for another 2 h or so. After incubation, absorption at 450 nm was measured at different times using an enzyme tagger. Colony formation was detected after incubation of 400 cells/well for 10–14 days in 6-well plates. The cells were fixed with methanol and stained with a solution of crystal violet. Clones containing at least 50 cells were counted using ImageJ (1.5a, National Institutes of Health, Bethesda, MD, USA).

### EdU assay

We used EdU Imaging Kits (APExBIO, Houston, TX, USA) based on the manufacturer’s instruction to perform this assay. The cells were plated in a 20 mm glass bottom cell culture dish and exposed to 10 μmol/L EdU for 4 hours. Following this, the cells were fixed at room temperature for 15 minutes with 4% paraformaldehyde in PBS followed by 0.5% Triton-100 in PBS. The click reaction solution, as configured, was subsequently introduced and incubated for 30 minutes at room temperature in dark conditions. Following three washes with PBS (3 minutes each), the cells were then incubated with a diluted solution of Hoechst 33342 (1:2000) at room temperature in the dark for 30 minutes. The images were obtained under laser confocal microscopy (Olympus, Tokyo, Japan), and the number of positive cells (red stained) was counted using ImageJ.

### Wound healing assay

Cells were seeded in 6-well plates (4 × 10^5^ cells/well). When the cell confluence reached 100%, the fused cells were scratched with a 200 μl pipette tip, the detached cells were washed off with PBS, and finally, pictures of the scratches were taken at 0 h and 24 h with an inverted microscope (Canon EOS 800D, Tokyo, Japan). ImageJ was used to count the migration distances in the selected fields.

### Transwell assay

After centrifugation of the cell suspension obtained by digestion, the upper medium was removed and the cells were resuspended in a serum-free medium; 200 μl (approximately 1 × 10^5^ cells) was inoculated into the upper chamber, and then 20% FBS containing culture (500 μl) was added to the lower chamber. After 24 hours of culture, ImageJ was used to count the number of migrating cells in the selected domain.

### Statistical analysis

The statistical analysis of bioinformatics was based on Perl language (Strawberry Perl 5.30.0.1) and R program (Version 4.2.1), and the experimental data were analyzed using ImageJ and GraphPad Prism 8.0. We used a *t*-test to compare the differences between the different groups. Error bars are mean ± SD, and *P* < 0.05 was considered statistically significant.

## Supplementary Materials

Supplementary Figures

Supplementary Table 1

Supplementary Tables 2 and 3
