# Supplementary material for: Identification and validation of a novel signature based on macrophage marker genes for predicting prognosis and drug response in kidney renal clear cell carcinoma by integrated analysis of single cell and bulk RNA sequencing
Source: Aging (Albany NY). 2024 Mar 20;16(6):5676–702. doi: 10.18632/aging.205671 (PMC11006469; doi:10.18632/aging.205671)
Supplement: Supplementary Figures [file aging-16-205671-s001.pdf]

SUPPLEMENTARY FIGURES

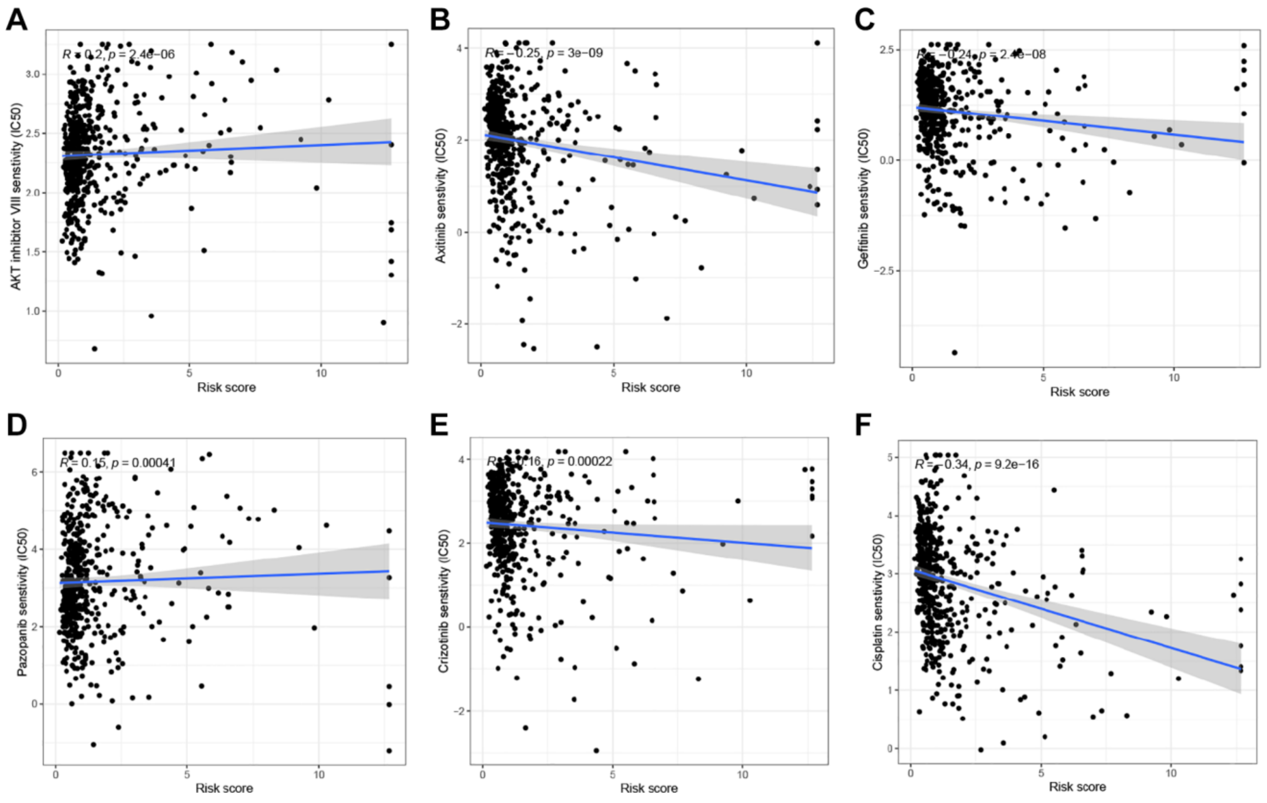

Supplementary Figure 1. Correlation analysis between risk score and IC50 of drugs. (**A**) AKT inhibitor, (**B**) Axitinib, (**C**) Gefitinib, (**D**) Pazopanib, (**E**) Crizotinib, and (**F**) Cisplatin.

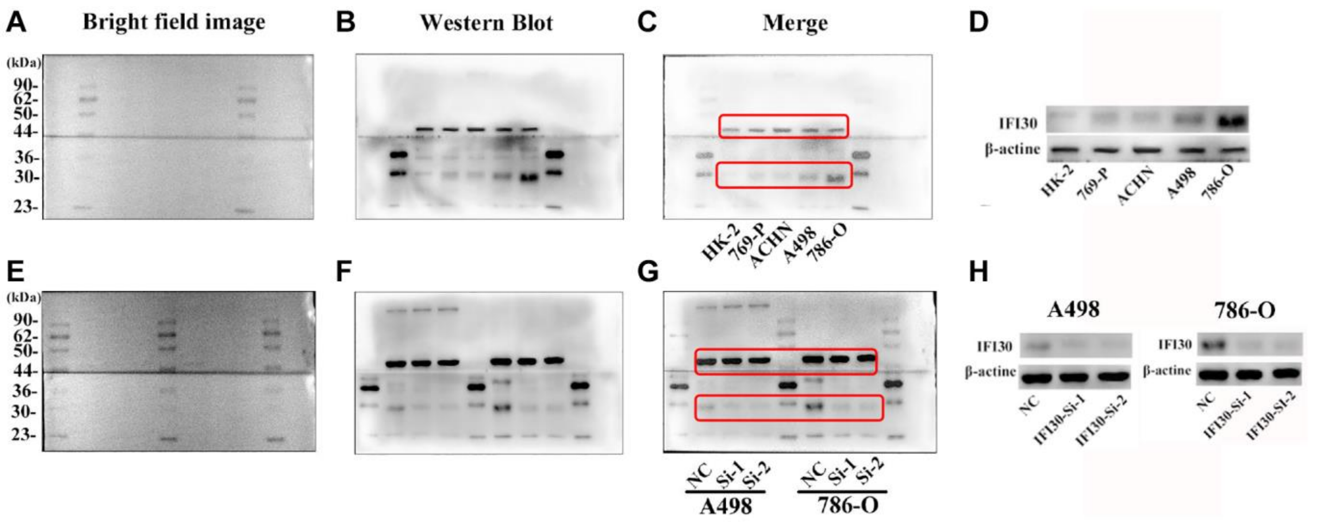

Supplementary Figure 2. Original images for Western blots and gels. The expression of IFI30 in cell lines: (**A**) Bright-field image, (**B**) Gels image, (**C**) Merge image, and (**D**) Cropped Western blots; Verification of the interference efficiency of IFI30 in A498 and 786-0: (**E**) Bright-field image, (**F**) Gels image, (**G**) Merge image, and (**H**) Cropped Western blots.
