# Supplementary material for: Identification and validation of a novel signature based on macrophage marker genes for predicting prognosis and drug response in kidney renal clear cell carcinoma by integrated analysis of single cell and bulk RNA sequencing
Source: Aging (Albany NY). 2024 Mar 20;16(6):5676–702. doi: 10.18632/aging.205671 (PMC11006469; doi:10.18632/aging.205671)
Supplement: Supplementary Table 1 [file aging-16-205671-s002.docx]

**Supplementary Table 1. Macrophage marker genes.**

| **P_val** | **Avg_log2FC** | **Pct.1** | **Pct.2** | **P_val_adj** | **Cluster** | **Gene** |
| --- | --- | --- | --- | --- | --- | --- |
| 0 | 3.581223 | 0.985 | 0.394 | 0 | Macrophage | C1QC |
| 1.38E-307 | 2.433861 | 0.988 | 0.296 | 2.12E-303 | Macrophage | MS4A6A |
| 6.81E-306 | 3.349764 | 0.993 | 0.626 | 1.04E-301 | Macrophage | C1QA |
| 3.78E-305 | 3.476592 | 0.991 | 0.606 | 5.78E-301 | Macrophage | C1QB |
| 2.69E-283 | 2.109968 | 0.971 | 0.36 | 4.12E-279 | Macrophage | HLA-DMB |
| 1.39E-280 | 2.213628 | 0.991 | 0.52 | 2.13E-276 | Macrophage | CD68 |
| 5.33E-278 | 1.954639 | 0.999 | 0.816 | 8.16E-274 | Macrophage | NPC2 |
| 2.14E-275 | 1.903862 | 1 | 0.873 | 3.27E-271 | Macrophage | GPX1 |
| 3.52E-274 | 1.960849 | 0.993 | 0.583 | 5.39E-270 | Macrophage | HLA-DMA |
| 5.73E-272 | 2.221202 | 0.998 | 0.757 | 8.77E-268 | Macrophage | HLA-DRB5 |
| 4.27E-268 | 2.340524 | 1 | 0.895 | 6.53E-264 | Macrophage | HLA-DRA |
| 1.24E-265 | 2.272606 | 0.854 | 0.142 | 1.90E-261 | Macrophage | TREM2 |
| 1.53E-262 | 2.139901 | 1 | 0.705 | 2.34E-258 | Macrophage | TYROBP |
| 1.68E-262 | 2.191903 | 0.999 | 0.828 | 2.57E-258 | Macrophage | HLA-DRB1 |
| 1.63E-260 | 2.168331 | 0.996 | 0.51 | 2.50E-256 | Macrophage | HLA-DQA1 |
| 7.41E-260 | 2.21043 | 0.999 | 0.809 | 1.13E-255 | Macrophage | HLA-DPA1 |
| 1.53E-259 | 1.963291 | 0.83 | 0.117 | 2.34E-255 | Macrophage | GPR34 |
| 5.45E-257 | 1.811429 | 0.985 | 0.631 | 8.34E-253 | Macrophage | FCGRT |
| 8.87E-257 | 3.203925 | 0.991 | 0.854 | 1.36E-252 | Macrophage | APOC1 |
| 7.11E-256 | 3.667221 | 0.973 | 0.91 | 1.09E-251 | Macrophage | APOE |
| 7.20E-255 | 2.013958 | 1 | 1 | 1.10E-250 | Macrophage | FTL |
| 4.92E-254 | 1.83228 | 0.928 | 0.199 | 7.53E-250 | Macrophage | MS4A7 |
| 1.09E-248 | 2.028811 | 1 | 0.858 | 1.67E-244 | Macrophage | HLA-DPB1 |
| 3.20E-248 | 1.973184 | 0.975 | 0.603 | 4.90E-244 | Macrophage | GRN |
| 3.48E-244 | 1.609169 | 0.885 | 0.186 | 5.32E-240 | Macrophage | LY86 |
| 6.87E-243 | 1.871206 | 1 | 0.969 | 1.05E-238 | Macrophage | CD74 |
| 1.49E-241 | 1.975106 | 0.851 | 0.166 | 2.28E-237 | Macrophage | MS4A4A |
| 8.22E-241 | 1.585638 | 0.804 | 0.121 | 1.26E-236 | Macrophage | LILRB4 |
| 3.23E-232 | 1.912195 | 0.988 | 0.599 | 4.94E-228 | Macrophage | HLA-DQB1 |
| 1.10E-230 | 2.095412 | 0.908 | 0.374 | 1.69E-226 | Macrophage | LIPA |
| 7.36E-228 | 2.087516 | 0.851 | 0.197 | 1.13E-223 | Macrophage | CD14 |
| 7.51E-222 | 1.563415 | 0.758 | 0.104 | 1.15E-217 | Macrophage | FCGR1A |
| 5.06E-217 | 1.683293 | 0.985 | 0.685 | 7.75E-213 | Macrophage | CTSB |
| 1.17E-211 | 1.574267 | 0.978 | 0.446 | 1.79E-207 | Macrophage | CTSS |
| 7.70E-210 | 1.544736 | 0.777 | 0.14 | 1.18E-205 | Macrophage | MSR1 |
| 2.70E-205 | 1.758919 | 0.994 | 0.874 | 4.13E-201 | Macrophage | PSAP |
| 2.72E-201 | 1.967408 | 0.724 | 0.125 | 4.15E-197 | Macrophage | VSIG4 |
| 7.74E-200 | 1.390527 | 0.866 | 0.245 | 1.18E-195 | Macrophage | CXCL16 |
| 5.61E-198 | 1.300134 | 0.777 | 0.142 | 8.58E-194 | Macrophage | LAIR1 |
| 6.79E-193 | 1.493135 | 0.896 | 0.284 | 1.04E-188 | Macrophage | MAFB |
| 2.85E-192 | 1.44364 | 0.824 | 0.204 | 4.36E-188 | Macrophage | FCGR2A |
| 1.19E-187 | 1.908686 | 0.859 | 0.287 | 1.81E-183 | Macrophage | CFD |
| 1.38E-186 | 1.406429 | 0.998 | 0.674 | 2.11E-182 | Macrophage | LAPTM5 |
| 6.19E-185 | 1.244787 | 0.664 | 0.091 | 9.46E-181 | Macrophage | SLC1A3 |
| 4.40E-183 | 1.314672 | 0.681 | 0.111 | 6.73E-179 | Macrophage | NPL |
| 6.18E-183 | 1.457361 | 0.71 | 0.118 | 9.46E-179 | Macrophage | IGSF6 |
| 7.45E-182 | 1.979561 | 0.64 | 0.089 | 1.14E-177 | Macrophage | FOLR2 |
| 7.35E-180 | 1.940765 | 0.993 | 0.883 | 1.12E-175 | Macrophage | CTSD |
| 5.12E-179 | 1.309792 | 0.993 | 0.499 | 7.84E-175 | Macrophage | AIF1 |
| 1.67E-177 | 1.168577 | 0.669 | 0.104 | 2.56E-173 | Macrophage | SLCO2B1 |
| 3.76E-177 | 1.959541 | 0.829 | 0.314 | 5.76E-173 | Macrophage | LGMN |
| 1.35E-175 | 1.685593 | 0.962 | 0.5 | 2.06E-171 | Macrophage | RGS1 |
| 1.97E-174 | 1.442386 | 0.939 | 0.369 | 3.01E-170 | Macrophage | FCGR3A |
| 3.83E-173 | 1.274482 | 0.914 | 0.385 | 5.87E-169 | Macrophage | CTSH |
| 6.14E-170 | 1.273597 | 0.995 | 0.579 | 9.40E-166 | Macrophage | FCER1G |
| 9.68E-169 | 1.481983 | 0.933 | 0.552 | 1.48E-164 | Macrophage | ASAH1 |
| 2.20E-168 | 1.259715 | 0.792 | 0.224 | 3.36E-164 | Macrophage | IL18 |
| 9.77E-168 | 1.311115 | 0.635 | 0.102 | 1.49E-163 | Macrophage | KCNMA1 |
| 9.73E-167 | 2.132028 | 0.759 | 0.255 | 1.49E-162 | Macrophage | GPNMB |
| 2.64E-166 | 1.392875 | 0.711 | 0.154 | 4.04E-162 | Macrophage | CPM |
| 3.17E-166 | 1.487389 | 0.899 | 0.423 | 4.84E-162 | Macrophage | AKR1B1 |
| 7.26E-162 | 1.183921 | 0.817 | 0.271 | 1.11E-157 | Macrophage | MFSD1 |
| 9.31E-162 | 1.238133 | 0.876 | 0.328 | 1.42E-157 | Macrophage | LY96 |
| 3.52E-159 | 1.148288 | 0.953 | 0.385 | 5.38E-155 | Macrophage | C1orf162 |
| 1.80E-158 | 1.354957 | 0.947 | 0.559 | 2.76E-154 | Macrophage | GLUL |
| 2.57E-158 | 1.245239 | 0.757 | 0.182 | 3.93E-154 | Macrophage | RNASE6 |
| 3.56E-155 | 1.028993 | 0.999 | 0.93 | 5.45E-151 | Macrophage | SAT1 |
| 9.72E-154 | 1.174702 | 0.984 | 0.644 | 1.49E-149 | Macrophage | RNASET2 |
| 3.84E-152 | 1.65668 | 0.838 | 0.407 | 5.88E-148 | Macrophage | PLD3 |
| 7.46E-142 | 1.153808 | 0.865 | 0.398 | 1.14E-137 | Macrophage | RNF130 |
| 5.21E-140 | 1.026232 | 0.62 | 0.119 | 7.97E-136 | Macrophage | ADAP2 |
| 1.40E-138 | 1.0631 | 0.727 | 0.186 | 2.15E-134 | Macrophage | IFI30 |
| 4.86E-138 | 1.4401 | 0.913 | 0.55 | 7.43E-134 | Macrophage | CAPG |
| 1.82E-137 | -1.70053 | 0.999 | 0.994 | 2.79E-133 | Macrophage | GAPDH |
| 1.80E-132 | 1.226376 | 0.831 | 0.411 | 2.75E-128 | Macrophage | CREG1 |
| 4.59E-132 | 1.017828 | 0.646 | 0.148 | 7.02E-128 | Macrophage | CD163 |
| 5.43E-131 | 1.053917 | 0.955 | 0.647 | 8.31E-127 | Macrophage | SDCBP |
| 8.70E-130 | 1.282081 | 0.461 | 0.046 | 1.33E-125 | Macrophage | SDS |
| 2.12E-126 | 1.081321 | 0.796 | 0.318 | 3.24E-122 | Macrophage | MARCKS |
| 9.26E-124 | -1.55854 | 0.958 | 0.962 | 1.42E-119 | Macrophage | ALDOA |
| 1.95E-122 | -1.34274 | 0.952 | 0.963 | 2.99E-118 | Macrophage | HINT1 |
| 2.89E-121 | -1.94636 | 0.886 | 0.907 | 4.43E-117 | Macrophage | MIF |
| 1.39E-118 | -2.42392 | 0.555 | 0.8 | 2.12E-114 | Macrophage | IL32 |
| 4.91E-117 | -1.43298 | 0.96 | 0.948 | 7.51E-113 | Macrophage | TPI1 |
| 5.82E-117 | 1.08248 | 0.501 | 0.088 | 8.91E-113 | Macrophage | OLR1 |
| 6.94E-115 | 1.092578 | 0.423 | 0.044 | 1.06E-110 | Macrophage | OTOA |
| 5.59E-114 | -1.56824 | 0.484 | 0.764 | 8.55E-110 | Macrophage | C12orf57 |
| 2.85E-110 | 1.785995 | 0.854 | 0.441 | 4.35E-106 | Macrophage | LYZ |
| 2.58E-109 | -1.41615 | 0.838 | 0.905 | 3.95E-105 | Macrophage | SEC61G |
| 9.29E-107 | 1.032224 | 0.759 | 0.314 | 1.42E-102 | Macrophage | CPVL |
| 9.70E-106 | 1.486889 | 0.864 | 0.526 | 1.48E-101 | Macrophage | LGALS3 |
| 9.47E-105 | 1.013301 | 0.567 | 0.14 | 1.45E-100 | Macrophage | FCGR2B |
| 2.46E-104 | 1.041246 | 0.643 | 0.193 | 3.76E-100 | Macrophage | AXL |
| 7.64E-104 | 1.070819 | 0.785 | 0.314 | 1.17E-99 | Macrophage | FAM26F |
| 8.99E-104 | 1.305695 | 0.647 | 0.192 | 1.38E-99 | Macrophage | RP11-1143G9.4 |
| 1.70E-103 | -1.82225 | 0.867 | 0.89 | 2.61E-99 | Macrophage | LDHA |
| 4.69E-101 | 1.003013 | 0.563 | 0.155 | 7.18E-97 | Macrophage | FUCA1 |
| 1.01E-100 | 1.059327 | 0.525 | 0.133 | 1.55E-96 | Macrophage | C2 |
| 2.00E-99 | -1.72741 | 0.576 | 0.758 | 3.06E-95 | Macrophage | DSTN |
| 1.75E-98 | 1.104367 | 0.871 | 0.495 | 2.68E-94 | Macrophage | CTSL |
| 5.21E-98 | -1.02404 | 0.994 | 0.994 | 7.97E-94 | Macrophage | COX7C |
| 9.15E-97 | -1.65307 | 0.122 | 0.525 | 1.40E-92 | Macrophage | PDLIM1 |
| 3.12E-95 | 1.095658 | 0.621 | 0.184 | 4.78E-91 | Macrophage | HLA-DQA2 |
| 9.04E-93 | -1.02423 | 0.863 | 0.917 | 1.38E-88 | Macrophage | SKP1 |
| 7.47E-89 | -1.28335 | 0.06 | 0.448 | 1.14E-84 | Macrophage | CNN3 |
| 1.36E-87 | -1.62398 | 0.264 | 0.593 | 2.08E-83 | Macrophage | OCIAD2 |
| 1.97E-87 | -1.18673 | 0.811 | 0.862 | 3.02E-83 | Macrophage | TBCA |
| 1.25E-84 | -1.30798 | 0.873 | 0.92 | 1.91E-80 | Macrophage | S100A10 |
| 1.57E-84 | -1.56598 | 0.742 | 0.824 | 2.40E-80 | Macrophage | LDHB |
| 4.76E-84 | -1.61829 | 0.175 | 0.532 | 7.28E-80 | Macrophage | C12orf75 |
| 1.95E-81 | -3.2297 | 0.337 | 0.614 | 2.98E-77 | Macrophage | CD24 |
| 2.86E-81 | -1.9338 | 0.115 | 0.48 | 4.38E-77 | Macrophage | CALD1 |
| 5.06E-81 | -2.58799 | 0.697 | 0.794 | 7.74E-77 | Macrophage | TIMP1 |
| 1.32E-77 | 1.482358 | 0.603 | 0.26 | 2.01E-73 | Macrophage | ACP5 |
| 6.10E-77 | -1.4891 | 0.235 | 0.553 | 9.33E-73 | Macrophage | NGFRAP1 |
| 1.08E-76 | -1.07598 | 0.617 | 0.782 | 1.65E-72 | Macrophage | RHOC |
| 2.20E-76 | 1.260765 | 0.586 | 0.226 | 3.36E-72 | Macrophage | GADD45G |
| 4.11E-76 | -1.19773 | 0.095 | 0.445 | 6.29E-72 | Macrophage | CAV2 |
| 4.19E-76 | -1.40194 | 0.093 | 0.448 | 6.41E-72 | Macrophage | IFITM1 |
| 5.94E-76 | -1.82724 | 0.258 | 0.584 | 9.08E-72 | Macrophage | CRIP1 |
| 1.88E-73 | -1.26177 | 0.332 | 0.624 | 2.88E-69 | Macrophage | PRDX2 |
| 3.87E-73 | -1.62906 | 0.141 | 0.479 | 5.92E-69 | Macrophage | CAV1 |
| 5.16E-71 | -1.61971 | 0.867 | 0.846 | 7.89E-67 | Macrophage | IFITM3 |
| 1.80E-70 | 1.012804 | 0.668 | 0.298 | 2.75E-66 | Macrophage | PLAUR |
| 1.19E-69 | -1.74101 | 0.254 | 0.552 | 1.82E-65 | Macrophage | TPM1 |
| 6.65E-69 | -1.14781 | 0.102 | 0.432 | 1.02E-64 | Macrophage | PRKCDBP |
| 3.04E-68 | -1.09075 | 0.664 | 0.771 | 4.65E-64 | Macrophage | TAGLN2 |
| 1.91E-67 | -2.49811 | 0.165 | 0.47 | 2.92E-63 | Macrophage | CXCL14 |
| 4.23E-67 | -1.22322 | 0.965 | 0.952 | 6.48E-63 | Macrophage | NPM1 |
| 6.15E-67 | 1.85852 | 0.763 | 0.477 | 9.41E-63 | Macrophage | SEPP1 |
| 2.10E-66 | -1.3653 | 0.104 | 0.421 | 3.21E-62 | Macrophage | COX7A1 |
| 2.38E-66 | -1.07689 | 0.82 | 0.879 | 3.64E-62 | Macrophage | PPDPF |
| 3.33E-66 | -1.53828 | 0.107 | 0.425 | 5.10E-62 | Macrophage | AKR1C3 |
| 5.37E-66 | -4.04922 | 0.376 | 0.608 | 8.22E-62 | Macrophage | IGFBP7 |
| 3.37E-63 | -1.58215 | 0.709 | 0.798 | 5.16E-59 | Macrophage | IFITM2 |
| 1.87E-62 | -1.19555 | 0.101 | 0.412 | 2.86E-58 | Macrophage | CRIP2 |
| 2.51E-62 | -1.09307 | 0.61 | 0.738 | 3.85E-58 | Macrophage | MZT2A |
| 3.11E-62 | 1.099189 | 0.766 | 0.439 | 4.76E-58 | Macrophage | HMOX1 |
| 3.51E-62 | 1.317788 | 0.853 | 0.662 | 5.37E-58 | Macrophage | GADD45B |
| 3.84E-62 | -1.15336 | 0.964 | 0.965 | 5.87E-58 | Macrophage | VIM |
| 4.61E-62 | -1.26053 | 0.86 | 0.881 | 7.06E-58 | Macrophage | ENO1 |
| 7.06E-59 | -1.38857 | 0.239 | 0.512 | 1.08E-54 | Macrophage | IGFBP4 |
| 2.93E-57 | -2.07614 | 0.387 | 0.59 | 4.48E-53 | Macrophage | KRT18 |
| 3.81E-57 | -1.14359 | 0.038 | 0.313 | 5.83E-53 | Macrophage | FSTL1 |
| 7.94E-57 | -2.35008 | 0.94 | 0.891 | 1.22E-52 | Macrophage | CRYAB |
| 1.81E-56 | -1.28803 | 0.319 | 0.552 | 2.77E-52 | Macrophage | S100A13 |
| 5.81E-55 | -1.04557 | 0.069 | 0.349 | 8.89E-51 | Macrophage | EGLN3 |
| 6.34E-55 | 1.368381 | 0.696 | 0.441 | 9.70E-51 | Macrophage | IER3 |
| 2.57E-53 | -2.84831 | 0.137 | 0.41 | 3.93E-49 | Macrophage | SPARC |
| 2.32E-52 | -1.72706 | 0.354 | 0.558 | 3.55E-48 | Macrophage | KRT8 |
| 3.14E-52 | -1.11455 | 0.763 | 0.82 | 4.80E-48 | Macrophage | LY6E |
| 1.22E-50 | -1.05215 | 0.773 | 0.816 | 1.87E-46 | Macrophage | PKM |
| 1.71E-50 | -1.37017 | 0.103 | 0.368 | 2.62E-46 | Macrophage | GNG11 |
| 1.38E-49 | -1.64626 | 0.279 | 0.511 | 2.11E-45 | Macrophage | BNIP3 |
| 1.42E-49 | -1.95483 | 0.271 | 0.494 | 2.17E-45 | Macrophage | MGST1 |
| 1.42E-48 | -2.10649 | 0.701 | 0.74 | 2.17E-44 | Macrophage | GPX3 |
| 3.20E-48 | -1.68192 | 0.243 | 0.477 | 4.89E-44 | Macrophage | CLU |
| 1.22E-47 | -1.61169 | 0.077 | 0.332 | 1.87E-43 | Macrophage | MYL9 |
| 4.85E-47 | -1.99578 | 0.401 | 0.568 | 7.42E-43 | Macrophage | PDZK1IP1 |
| 6.52E-47 | -1.18535 | 0.374 | 0.58 | 9.98E-43 | Macrophage | LINC00152 |
| 9.17E-47 | -2.09347 | 0.636 | 0.686 | 1.40E-42 | Macrophage | CYB5A |
| 1.92E-46 | -1.0562 | 0.769 | 0.792 | 2.93E-42 | Macrophage | HMGN3 |
| 1.94E-46 | -3.4074 | 0.263 | 0.498 | 2.97E-42 | Macrophage | IGKC |
| 2.39E-45 | -1.43088 | 0.029 | 0.255 | 3.66E-41 | Macrophage | TM4SF1 |
| 5.48E-45 | 2.536532 | 0.663 | 0.431 | 8.38E-41 | Macrophage | CCL3 |
| 8.00E-44 | -2.62834 | 0.374 | 0.549 | 1.22E-39 | Macrophage | FXYD2 |
| 1.04E-43 | -1.01784 | 0.451 | 0.596 | 1.60E-39 | Macrophage | CYSTM1 |
| 3.92E-43 | -1.10549 | 0.05 | 0.278 | 6.00E-39 | Macrophage | BHMT |
| 5.98E-43 | -1.07354 | 0.177 | 0.416 | 9.15E-39 | Macrophage | CKB |
| 6.21E-43 | -1.06334 | 0.011 | 0.215 | 9.50E-39 | Macrophage | AKR1C1 |
| 6.37E-43 | -1.02882 | 0.278 | 0.499 | 9.74E-39 | Macrophage | NDRG1 |
| 2.48E-42 | -1.52044 | 0.431 | 0.579 | 3.80E-38 | Macrophage | ANXA4 |
| 2.79E-42 | 1.077527 | 0.447 | 0.198 | 4.26E-38 | Macrophage | CD83 |
| 4.02E-42 | -1.55561 | 0.07 | 0.298 | 6.15E-38 | Macrophage | FHL1 |
| 4.14E-42 | -1.16793 | 0.515 | 0.648 | 6.33E-38 | Macrophage | NBEAL1 |
| 8.06E-42 | -1.1481 | 0.086 | 0.317 | 1.23E-37 | Macrophage | CMBL |
| 1.75E-41 | -1.33677 | 0.106 | 0.344 | 2.68E-37 | Macrophage | MT1E |
| 5.86E-41 | -1.0465 | 0.194 | 0.426 | 8.97E-37 | Macrophage | APP |
| 8.72E-41 | -1.36651 | 0.045 | 0.261 | 1.33E-36 | Macrophage | FAM134B |
| 1.61E-40 | -1.62102 | 0.392 | 0.552 | 2.46E-36 | Macrophage | SNHG25 |
| 4.06E-40 | -1.15787 | 0.106 | 0.337 | 6.20E-36 | Macrophage | HILPDA |
| 6.17E-38 | 1.518971 | 0.4 | 0.184 | 9.44E-34 | Macrophage | CXCL3 |
| 8.67E-38 | -1.60933 | 0.053 | 0.26 | 1.33E-33 | Macrophage | TPM2 |
| 1.29E-37 | -1.29626 | 0.1 | 0.317 | 1.98E-33 | Macrophage | IGFBP6 |
| 2.64E-37 | -1.52226 | 0.363 | 0.516 | 4.04E-33 | Macrophage | SELM |
| 1.64E-36 | -2.65027 | 0.141 | 0.351 | 2.52E-32 | Macrophage | IGFBP3 |
| 1.65E-36 | -1.95244 | 0.692 | 0.696 | 2.52E-32 | Macrophage | NNMT |
| 3.12E-36 | -1.00396 | 0.798 | 0.793 | 4.77E-32 | Macrophage | CIB1 |
| 3.90E-36 | 2.124131 | 0.498 | 0.29 | 5.96E-32 | Macrophage | CXCL8 |
| 4.77E-36 | -1.08139 | 0.189 | 0.403 | 7.29E-32 | Macrophage | C19orf33 |
| 3.57E-34 | -1.32184 | 0.049 | 0.24 | 5.47E-30 | Macrophage | PRSS23 |
| 6.59E-33 | -1.23619 | 0.203 | 0.402 | 1.01E-28 | Macrophage | SPON2 |
| 9.66E-33 | -1.21502 | 0.1 | 0.298 | 1.48E-28 | Macrophage | C11orf54 |
| 4.90E-32 | -2.56475 | 0.812 | 0.771 | 7.50E-28 | Macrophage | NDUFA4L2 |
| 1.90E-31 | -1.0592 | 0.411 | 0.576 | 2.91E-27 | Macrophage | TSC22D1 |
| 2.83E-31 | -1.10083 | 0.192 | 0.393 | 4.32E-27 | Macrophage | ANGPTL4 |
| 5.05E-31 | -1.18804 | 0.052 | 0.231 | 7.72E-27 | Macrophage | COL6A2 |
| 1.55E-30 | -2.1363 | 0.508 | 0.657 | 2.37E-26 | Macrophage | NKG7 |
| 4.96E-30 | -1.64819 | 0.052 | 0.224 | 7.60E-26 | Macrophage | PTGDS |
| 1.55E-29 | -1.19166 | 0.056 | 0.23 | 2.37E-25 | Macrophage | S100A1 |
| 5.00E-29 | 1.369017 | 0.617 | 0.387 | 7.65E-25 | Macrophage | CCL3L3 |
| 2.36E-27 | 1.214572 | 0.345 | 0.166 | 3.61E-23 | Macrophage | IL1B |
| 5.25E-27 | -1.53271 | 0.196 | 0.381 | 8.04E-23 | Macrophage | ZNF90 |
| 5.48E-27 | -2.5947 | 0.203 | 0.363 | 8.39E-23 | Macrophage | FABP7 |
| 1.76E-26 | -1.21627 | 0.214 | 0.39 | 2.69E-22 | Macrophage | HIST1H4C |
| 1.25E-25 | -1.67789 | 0.09 | 0.251 | 1.92E-21 | Macrophage | GSTA2 |
| 2.41E-25 | -1.24022 | 0.138 | 0.309 | 3.68E-21 | Macrophage | CD3E |
| 4.86E-25 | -1.0169 | 0.252 | 0.414 | 7.43E-21 | Macrophage | MIR4435-2HG |
| 6.70E-25 | -1.01757 | 0.129 | 0.3 | 1.03E-20 | Macrophage | C1R |
| 1.03E-24 | -1.22365 | 0.067 | 0.222 | 1.58E-20 | Macrophage | KRT19 |
| 2.49E-24 | -3.0553 | 0.313 | 0.44 | 3.81E-20 | Macrophage | GSTA1 |
| 5.00E-24 | -1.69573 | 0.282 | 0.421 | 7.65E-20 | Macrophage | LGALS2 |
| 6.97E-24 | -1.54325 | 0.236 | 0.398 | 1.07E-19 | Macrophage | CST7 |
| 3.26E-23 | -1.11503 | 0.093 | 0.251 | 4.98E-19 | Macrophage | GZMH |
| 6.33E-23 | -1.26875 | 0.212 | 0.37 | 9.68E-19 | Macrophage | CCND1 |
| 2.91E-22 | -1.33886 | 0.238 | 0.403 | 4.45E-18 | Macrophage | TRBC2 |
| 4.01E-21 | 1.155669 | 0.433 | 0.262 | 6.14E-17 | Macrophage | CXCL2 |
| 4.09E-21 | -1.2307 | 0.124 | 0.28 | 6.26E-17 | Macrophage | CYR61 |
| 6.82E-21 | -1.01162 | 0.194 | 0.355 | 1.04E-16 | Macrophage | TUBA4A |
| 3.12E-20 | -4.58813 | 0.143 | 0.293 | 4.77E-16 | Macrophage | IGLC2 |
| 8.39E-20 | -1.11704 | 0.088 | 0.229 | 1.28E-15 | Macrophage | PLAC8 |
| 1.15E-19 | -1.24103 | 0.136 | 0.278 | 1.75E-15 | Macrophage | WFDC2 |
| 2.16E-19 | -1.19921 | 0.227 | 0.366 | 3.30E-15 | Macrophage | C10orf10 |
| 4.46E-18 | -1.23892 | 0.092 | 0.216 | 6.83E-14 | Macrophage | NAT8 |
| 5.72E-18 | -1.21484 | 0.138 | 0.267 | 8.75E-14 | Macrophage | SMIM24 |
| 8.63E-18 | -1.41624 | 0.092 | 0.215 | 1.32E-13 | Macrophage | MIOX |
| 2.25E-17 | -1.49953 | 0.263 | 0.398 | 3.44E-13 | Macrophage | CD3D |
| 2.43E-17 | -1.80082 | 0.2 | 0.332 | 3.72E-13 | Macrophage | GZMA |
| 3.27E-17 | -1.33659 | 0.169 | 0.305 | 5.00E-13 | Macrophage | TRBC1 |
| 6.99E-15 | -1.60906 | 0.134 | 0.256 | 1.07E-10 | Macrophage | GZMB |
| 1.56E-14 | -1.85447 | 0.333 | 0.45 | 2.39E-10 | Macrophage | GNLY |
| 1.60E-14 | -1.20332 | 0.756 | 0.717 | 2.45E-10 | Macrophage | RARRES2 |
| 5.12E-14 | -1.10098 | 0.137 | 0.25 | 7.84E-10 | Macrophage | CD2 |
| 5.15E-14 | -1.04018 | 0.299 | 0.401 | 7.88E-10 | Macrophage | RAC2 |
| 3.11E-13 | -1.22067 | 0.473 | 0.5 | 4.77E-09 | Macrophage | ATP1B1 |
| 9.00E-13 | -1.32245 | 0.471 | 0.571 | 1.38E-08 | Macrophage | CD52 |
| 1.07E-11 | -1.55962 | 0.178 | 0.274 | 1.64E-07 | Macrophage | TRAC |
| 5.41E-11 | -1.89523 | 0.496 | 0.556 | 8.28E-07 | Macrophage | CCL5 |
| 2.66E-10 | -1.12291 | 0.153 | 0.247 | 4.06E-06 | Macrophage | LTB |
| 7.67E-10 | -1.19565 | 0.621 | 0.605 | 1.17E-05 | Macrophage | SPP1 |
| 2.84E-09 | -1.00238 | 0.201 | 0.296 | 4.34E-05 | Macrophage | KLRB1 |
| 5.57E-09 | -1.20042 | 0.25 | 0.327 | 8.52E-05 | Macrophage | CD7 |
| 7.68E-09 | -1.27793 | 0.273 | 0.349 | 0.000118 | Macrophage | FN1 |
| 6.08E-07 | -3.37577 | 0.169 | 0.234 | 0.009306 | Macrophage | MGP |
