# Supplementary material for: Identification and validation of a novel signature based on macrophage marker genes for predicting prognosis and drug response in kidney renal clear cell carcinoma by integrated analysis of single cell and bulk RNA sequencing
Source: Aging (Albany NY). 2024 Mar 20;16(6):5676–702. doi: 10.18632/aging.205671 (PMC11006469; doi:10.18632/aging.205671)
Supplement: Supplementary Tables 2 and 3 [file aging-16-205671-s003.pdf]

SUPPLEMENTARY TABLES

Supplementary Table 2. The results of univariate regression analysis of the training set.

Gene	HR	HR.95L	HR.95H	*p*-value
HLA-DRA	0.847195	0.718101	0.999496	0.049305
GPR34	0.825701	0.701832	0.971433	0.020919
FCGRT	0.538468	0.38667	0.749859	0.000249
LIPA	0.707475	0.587212	0.852368	0.000272
FCGR1A	1.25263	1.005402	1.560652	0.044644
LGMN	0.742707	0.584154	0.944296	0.015192
CTSH	0.529963	0.389447	0.721177	5.36E-05
ASAH1	0.600399	0.480702	0.7499	6.89E-06
KCNMA1	0.759948	0.627371	0.92054	0.00501
CPM	0.689651	0.56329	0.844359	0.00032
MFSD1	0.695739	0.535894	0.903261	0.006452
LY96	1.322334	1.058618	1.651745	0.01382
SAT1	1.495539	1.074718	2.081138	0.016969
RNASET2	1.284705	1.094293	1.508249	0.002207
RNF130	0.575174	0.36182	0.914335	0.019354
IFI30	1.667812	1.305568	2.130565	4.24E-05
CREG1	0.760087	0.604187	0.956214	0.019168
HINT1	0.639886	0.46408	0.882291	0.006448
OTOA	0.223497	0.088439	0.564811	0.001537
LYZ	0.858697	0.773004	0.95389	0.004511
CPVL	0.812144	0.696391	0.947138	0.007996
FUCA1	0.462551	0.344102	0.621774	3.25E-07
C2	1.201378	1.032771	1.397511	0.017412
DSTN	0.505553	0.364789	0.700634	4.19E-05
CTSL	0.703532	0.521607	0.948909	0.021251
COX7C	0.4382	0.276483	0.694507	0.000446
SKP1	0.500874	0.344387	0.728468	0.000297
CNN3	0.6132	0.469538	0.800818	0.00033
OCIAD2	0.749662	0.593892	0.946288	0.015329
CD24	0.726255	0.615589	0.856816	0.000149
TIMP1	1.61787	1.32968	1.968521	1.53E-06
ACP5	0.771911	0.66292	0.898822	0.000858
PRDX2	0.627713	0.494551	0.796729	0.000129
PLAUR	1.633876	1.334906	1.999803	1.92E-06
TAGLN2	1.68683	1.139787	2.496428	0.008945
CXCL14	0.87094	0.804909	0.942388	0.000592
NPM1	0.658108	0.499739	0.866665	0.002894
MZT2A	1.314675	1.035193	1.669611	0.024857
HMOX1	0.837856	0.722273	0.971934	0.0195
GADD45B	1.245944	1.01339	1.531865	0.036965
ENO1	0.774124	0.63184	0.948448	0.013483
CRYAB	0.839357	0.723025	0.974406	0.021415
IER3	1.263574	1.065898	1.497909	0.007035
BNIP3	0.740863	0.625356	0.877705	0.000524

GPX3	0.772442	0.653862	0.912526	0.002394
PDZK1IP1	0.892691	0.814844	0.977975	0.014754
CYB5A	0.671991	0.559981	0.806407	1.93E-05
HMGN3	1.414896	1.029752	1.944092	0.032289
IGKC	1.12356	1.027283	1.228859	0.010807
FXYD2	0.678646	0.551242	0.835495	0.000258
CYSTM1	0.544908	0.380046	0.781286	0.000958
BHMT	0.836942	0.773843	0.905187	8.56E-06
AKR1C1	0.696075	0.58204	0.832452	7.22E-05
NDRG1	0.815593	0.685516	0.970353	0.021479
ANXA4	0.793181	0.65747	0.956903	0.015517
CD83	0.759306	0.619041	0.931352	0.00823
NBEAL1	0.632333	0.488186	0.819042	0.000516
CMBL	0.685989	0.590739	0.796597	7.75E-07
APP	0.739861	0.589151	0.929124	0.009528
SNHG25	1.276438	1.060417	1.536465	0.009878
CXCL3	1.443355	1.164116	1.789575	0.000822
CIB1	1.892944	1.345897	2.662343	0.000245
CXCL8	1.161953	1.031647	1.308717	0.013385
PRSS23	0.802165	0.656203	0.980594	0.031461
SPON2	1.243939	1.062988	1.455692	0.006497
C11orf54	0.586138	0.481123	0.714076	1.14E-07
TSC22D1	0.654867	0.517172	0.829224	0.00044
COL6A2	1.436221	1.191957	1.730541	0.000141
GSTA2	0.878579	0.816193	0.945733	0.000572
C1R	1.323955	1.13433	1.545279	0.000374
GSTA1	0.896215	0.831721	0.96571	0.004032
LGALS2	0.815218	0.745842	0.891047	6.73E-06
CCND1	0.814273	0.702372	0.944001	0.00645
CXCL2	1.254956	1.089415	1.445651	0.001652
IGLC2	1.148435	1.044945	1.262175	0.004073
NAT8	0.837271	0.782564	0.895803	2.58E-07
SMIM24	0.714575	0.651167	0.784157	1.35E-12
MIOX	0.839187	0.772443	0.911698	3.38E-05
ATP1B1	0.740798	0.608552	0.901784	0.002787
CD7	1.341999	1.117654	1.611377	0.001623

Supplementary Table 3. Immune cell infiltration with different algorithms.

Immune	cor	*p*-value
B cell_TIMER	–0.1827	2.20E-05
T cell CD4+_TIMER	0.21787	3.79E-07
Macrophage_TIMER	–0.10076	0.019979
B cell naive_CIBERSORT	–0.19438	6.18E-06
B cell memory_CIBERSORT	0.170783	7.41E-05
B cell plasma_CIBERSORT	–0.11441	0.008197
T cell CD8+_CIBERSORT	0.152013	0.000429
T cell CD4+ memory resting_CIBERSORT	–0.08979	0.038238
T cell CD4+ memory activated_CIBERSORT	0.172982	5.96E-05
T cell follicular helper_CIBERSORT	0.306198	4.94E-13
T cell regulatory (Tregs)_CIBERSORT	0.38537	2.58E-20
T cell gamma delta_CIBERSORT	–0.08658	0.045723
Monocyte_CIBERSORT	–0.13665	0.001566
Macrophage M0_CIBERSORT	0.174084	5.33E-05
Macrophage M2_CIBERSORT	–0.15967	0.000214
Mast cell activated_CIBERSORT	–0.16656	0.000112
Eosinophil_CIBERSORT	–0.10282	0.01757
B cell naive_CIBERSORT-ABS	–0.15063	0.000484
B cell memory_CIBERSORT-ABS	0.16798	9.75E-05
T cell CD8+_CIBERSORT-ABS	0.14724	0.00065
T cell CD4+ memory activated_CIBERSORT-ABS	0.172908	6.00E-05
T cell follicular helper_CIBERSORT-ABS	0.285577	1.84E-11
T cell regulatory (Tregs)_CIBERSORT-ABS	0.378113	1.47E-19
NK cell activated_CIBERSORT-ABS	0.114887	0.007932
Macrophage M0_CIBERSORT-ABS	0.171999	6.57E-05
Mast cell activated_CIBERSORT-ABS	–0.15573	0.000308
B cell_QUANTISEQ	0.134365	0.001878
Macrophage M1_QUANTISEQ	0.317981	5.48E-14
Monocyte_QUANTISEQ	0.237825	2.73E-08
Neutrophil_QUANTISEQ	–0.53793	2.61E-41
NK cell_QUANTISEQ	–0.09436	0.029387
T cell CD4+ (non-regulatory) QUANTISEQ	–0.34252	4.07E-16
T cell CD8+_QUANTISEQ	0.216537	4.48E-07
T cell regulatory (Tregs)_QUANTISEQ	0.258603	1.36E-09
uncharacterized cell_QUANTISEQ	0.111849	0.009758
T cell CD8+_MCPCOUNTER	0.107228	0.013253
cytotoxicity score_MCPCOUNTER	0.154345	0.000348
Myeloid dendritic cell_MCPCOUNTER	–0.17014	7.90E-05
Neutrophil_MCPCOUNTER	–0.40675	1.18E-22
Endothelial cell_MCPCOUNTER	–0.22413	1.70E-07

Cancer associated fibroblast_MCPCOUNTER	0.364531	3.40E-18
Myeloid dendritic cell activated_XCELL	0.237836	2.73E-08
B cell_XCELL	0.22524	1.48E-07
T cell CD4+ naive_XCELL	0.267986	3.22E-10
T cell CD4+ central memory_XCELL	0.137421	0.001471
T cell CD4+ effector memory_XCELL	0.115505	0.007601
T cell CD8+ naive_XCELL	0.106782	0.013644
T cell CD8+_XCELL	0.172948	5.98E-05
T cell CD8+ central memory_XCELL	0.104476	0.015824
T cell CD8+ effector memory_XCELL	0.201953	2.60E-06
Class-switched memory B cell_XCELL	0.18312	2.10E-05
Common lymphoid progenitor_XCELL	–0.16368	0.000147
Myeloid dendritic cell_XCELL	0.096855	0.025346
Endothelial cell_XCELL	–0.12138	0.005013
Cancer associated fibroblast_XCELL	0.133251	0.00205
Granulocyte-monocyte progenitor_XCELL	–0.10022	0.020666
Hematopoietic stem cell_XCELL	–0.24757	6.93E-09
Macrophage M1_XCELL	0.161886	0.000174
Macrophage M2_XCELL	–0.15247	0.000412
Mast cell_XCELL	–0.14739	0.000641
Monocyte_XCELL	0.159148	0.000225
B cell naive_XCELL	0.124741	0.003922
Neutrophil_XCELL	–0.08897	0.040048
T cell NK_XCELL	0.54204	4.92E-42
Plasmacytoid dendritic cell_XCELL	0.129805	0.002678
B cell plasma_XCELL	0.086633	0.045594
T cell CD4+ Th1_XCELL	0.345715	2.08E-16
T cell CD4+ Th2_XCELL	0.11836	0.006224
immune score_XCELL	0.205747	1.67E-06
microenvironment score_XCELL	0.245138	9.82E-09
B cell_EPIC	–0.14113	0.001087
Cancer associated fibroblast_EPIC	0.359103	1.15E-17
T cell CD4+_EPIC	–0.33787	1.07E-15
T cell CD8+_EPIC	–0.22823	9.97E-08
Endothelial cell_EPIC	–0.23637	3.34E-08
Macrophage_EPIC	0.183773	1.96E-05
NK cell_EPIC	0.256331	1.92E-09
uncharacterized cell_EPIC	0.138785	0.001318
